# Human Inborn Errors of Immunity: 2022 Update on the Classification from the International Union of Immunological Societies Expert Committee

**DOI:** 10.1007/s10875-022-01289-3

**Published:** 2022-06-24

**Authors:** Stuart G. Tangye, Waleed Al-Herz, Aziz Bousfiha, Charlotte Cunningham-Rundles, Jose Luis Franco, Steven M. Holland, Christoph Klein, Tomohiro Morio, Eric Oksenhendler, Capucine Picard, Anne Puel, Jennifer Puck, Mikko R. J. Seppänen, Raz Somech, Helen C. Su, Kathleen E. Sullivan, Troy R. Torgerson, Isabelle Meyts

**Affiliations:** 1 Garvan Institute of Medical Research, Darlinghurst, Sydney, NSW 2010 Australia; 2 St Vincent’s Clinical School, Faculty of Medicine & Health, UNSW Sydney, Darlinghurst, NSW Australia; 3 Department of Pediatrics, Faculty of Medicine, Kuwait University, Kuwait City, Kuwait; 4 Laboratoire d’Immunologie Clinique, d’Inflammation et d’Allergy LICIA Clinical Immunology Unit, Casablanca Children’s Hospital, Ibn Rochd Medical School, King Hassan II University, Casablanca, Morocco; 5 Departments of Medicine and Pediatrics, Mount Sinai School of Medicine, New York, NY USA; 6 Grupo de Inmunodeficiencias Primarias, Facultad de Medicina, Universidad de Antioquia UdeA, Medellin, Colombia; 7 Laboratory of Clinical Immunology & Microbiology, National Institute of Allergy and Infectious Diseases, National Institutes of Health, Bethesda, MD USA; 8 Dr von Hauner Children’s Hospital, Ludwig-Maximilians-University Munich, Munich, Germany; 9 Department of Pediatrics and Developmental Biology, Tokyo Medical and Dental University, Tokyo, Japan; 10 Department of Clinical Immunology, Hôpital Saint-Louis, APHP, Université Paris Diderot, Sorbonne Paris Cité, Paris, France; 11 Study Center for Primary Immunodeficiencies, Necker Hospital for Sick Children, APHP, Paris, France; 12 Laboratory of Lymphocyte Activation and Susceptibility to EBV, INSERM UMR1163, Imagine Institute, Necker Hospital for Sick Children, Université Paris Cité, Paris, France; 13 Laboratory of Human Genetics of Infectious Diseases, INSERM U1163, Necker Hospital, 75015 Paris, France; 14 Université Paris Cité, Imagine Institute, 75015 Paris, France; 15 Department of Pediatrics, University of California San Francisco and UCSF Benioff Children’s Hospital, San Francisco, CA USA; 16 Adult Immunodeficiency Unit, Infectious Diseases, Inflammation Center and Rare Diseases Center, Children’s Hospital, University of Helsinki and Helsinki University Hospital, Helsinki, Finland; 17 Pediatric Department and Immunology Unit, Sheba Medical Center, Tel Aviv, Israel; 18 Division of Allergy Immunology, Department of Pediatrics, Children’s Hospital of Philadelphia, University of Pennsylvania Perelman School of Medicine, Philadelphia, PA USA; 19 Allen Institute for Immunology, Seattle, WA USA; 20 Department of Immunology and Microbiology, Laboratory for Inborn Errors of Immunity, Department of Pediatrics, University Hospitals Leuven and KU Leuven, 3000 Leuven, Belgium

**Keywords:** Inborn errors of immunity, immune dysregulation, primary immunodeficiencies, autoinflammatory disorders, IUIS Committee update

## Abstract

We report the updated classification of inborn errors of immunity, compiled by the International Union of Immunological Societies Expert Committee. This report documents the key clinical and laboratory features of 55 novel monogenic gene defects, and 1 phenocopy due to autoantibodies, that have either been discovered since the previous update (published January 2020) or were characterized earlier but have since been confirmed or expanded in subsequent studies. While variants in additional genes associated with immune diseases have been reported in the literature, this update includes only those that the committee assessed that reached the necessary threshold to represent novel inborn errors of immunity. There are now a total of 485 inborn errors of immunity. These advances in discovering the genetic causes of human immune diseases continue to significantly further our understanding of molecular, cellular, and immunological mechanisms of disease pathogenesis, thereby simultaneously enhancing immunological knowledge and improving patient diagnosis and management. This report is designed to serve as a resource for immunologists and geneticists pursuing the molecular diagnosis of individuals with heritable immunological disorders and for the scientific dissection of cellular and molecular mechanisms underlying monogenic and related human immune diseases.

**Supplementary Information:**

The online version contains supplementary material available at 10.1007/s10875-022-01289-3.

## Introduction

Inborn errors of immunity (IEI) are caused by damaging germline variants in single genes. IEI present clinically as increased susceptibility to infections, autoimmunity, autoinflammatory diseases, allergy, bone marrow failure, and/or malignancy. While individually rare, the aggregated number of individuals with an IEI represents a significant health burden [1]. Genetic variants cause disease by altering the encoded gene product, such as by abolishing or reducing protein expression and function (null/hypomorphic) or modifying the protein to acquire gain-of-function (GOF) [2–5]. Mechanisms of disease in IEI depend on the nature of the variant as well as the mode of inheritance. Thus, monoallelic variants can cause disease by haploinsufficiency, negative dominance, or GOF. In contrast, biallelic genetic lesions (homozygous, compound heterozygous) cause autosomal recessive (AR) traits by loss of expression, loss of function (LOF), GOF, or even neomorphic function of the encoded protein, while X-linked recessive traits arise from LOF or GOF variants on the X chromosome, either in hemizygosity in males, or homozygous state in females.

The fact that some monogenic variants are pathogenic clearly highlights the non-redundant and fundamental roles of individual genes and proteins, and associated pathways and cell types, in the development and function of leukocytes and non-hematopoietic cells that contribute to immune homeostasis and host defense [6, 7]. Thus, IEI represent an elegant model linking defined monogenic defects with clinical phenotypes of immune dysregulation. IEI have also revealed mechanisms of disease pathogenesis in, and enabled the implementation of gene- or pathway-specific therapies for the treatment of, rare and common conditions and established fundamental aspects of human immunology [8–10]. Thus, the study of IEI has enabled profound advances in molecular medicine and human biology.

**Fig. 1:**
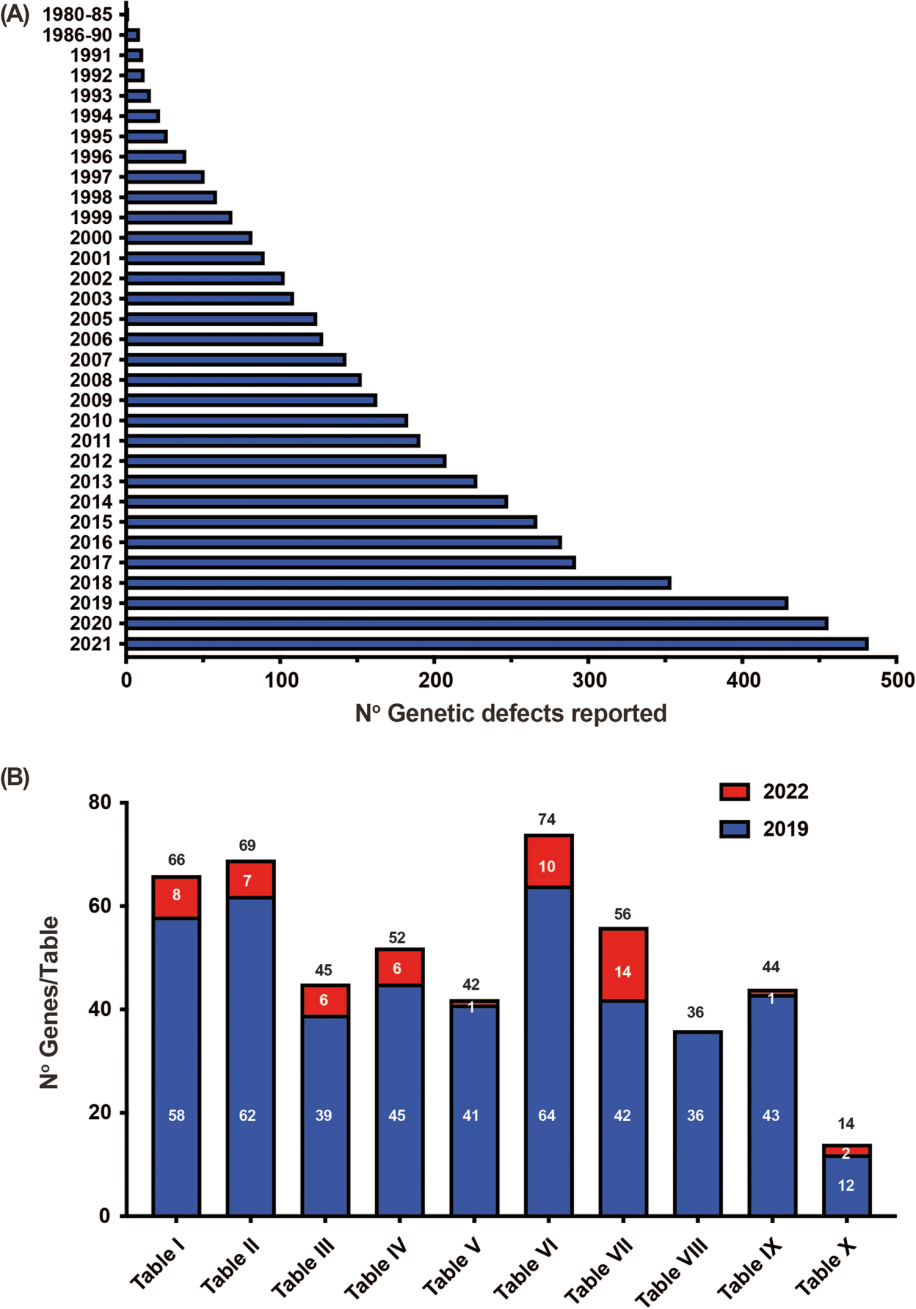
Accumulative discovery of novel inborn errors of immunity: 1980–2022. (A) The number of genetic defects underlying monogenic immune disorders as reported in the indicated year. (B) The number of pathogenic variants listed in each Table of the IUIS IEI committee 2022 report. The numbers in each column correspond to the number of genes reported in the 2019 IUIS update (blue bars) [4, 5], the number of new genes for each Table contained in this report (red bars), and the total number of genes for each Table (black number). Note: The 14 conditions listed for Table 10 are either phenocopies of germline IEI due to somatic variants or neutralizing autoAbs. Somatic variants in *UBA1* are also listed in Table 10, although there is currently no IEI resulting from germline *UBA1* variants [97]

Since 1970, an international expert committee comprising pediatric and adult clinical immunologists, clinician/scientists and researchers in basic immunology — initially under the auspices of the World Health Organization and currently the International Union of Immunological Societies (IUIS) — has provided the clinical and research communities with an update of genetic causes of immune deficiency and dysregulation https://iuis.org/committees/iei/ (Fig. 1A).

IEI are currently categorized into 10 Tables, with subtables segregating groups of disorders into overlapping phenotypes. These tables describe the following: combined immunodeficiencies (Table 1, 3 subtables); combined immunodeficiencies with syndromic features (Table 2; 9 subtables); predominantly antibody deficiencies (Table 3; 3 subtables); diseases of immune dysregulation (Table 4; 7 subtables); congenital defects of phagocytes (Table 5; 4 subtables); defects in intrinsic and innate immunity (Table 6; 9 subtables); autoinflammatory diseases (Table 7; 3 subtables); complement deficiencies (Table 8); bone marrow failure (Table 9), and phenocopies of inborn errors of immunity (Table 10) (Fig. 1B) [5].

The committee strives to publish an updated report approximately every 2 years to consolidate advances and catalog current IEIs (Fig. 1A) [5]. While COVID-19 has delayed producing this report in the desired timeframe, it has also uncovered several new IEI — some of these are highlighted below. Many genetic variants related to IEI have been reported recently. Rather than including every candidate gene reported in the peer-reviewed scientific literature, the committee applies stringent criteria to classify gene defects as novel causes of IEI [11]. These criteria include:

1. The patient’s candidate genotype is monogenic and does not occur in individuals without the clinical phenotype (acknowledging that some conditions have incomplete penetrance).
2. Experimental studies establish that the genetic variant impairs, destroys, or alters expression or function of the gene product.
3. The causal relationship between the candidate genotype and the clinical phenotype must be confirmed via a relevant cellular phenotype, including — where possible — rescue of a functional defect [11].

These criteria can be met by publication of multiple cases from unrelated kindreds, including detailed immunologic data, or publication of very few — even single — cases for whom compelling mechanistic data are provided, often revealed from complementary studies in animal or cell culture models. We also considered whether sufficient justification was provided to exclude alternative candidate gene variants identified in single cases, the depth of the clinical descriptions of affected individuals, and the level of immune and mechanistic characterization. This 2022 update and the accompanying “Phenotypical IUIS Classification” publications are intended as resources for clinicians and researchers, as well as guiding the design of panels used for targeted gene sequencing to facilitate genetic diagnoses of IEI. Here, we summarize data on the genetic cause of 55 novel IEI, and 1 phenocopy due to autoantibodies, that have been assessed since the previous update [5] (Supplementary Table 1). Remarkably, 15 of the 55 novel IEI have come from the identification and extensive work-up of single patients. Two themes that are expanded in this new set of genes are narrow infection susceptibility and immune dysregulation, which collectively account for over half of the phenotypes associated with these new genetic etiologies of IEI. This paper increases the number of known genetic defects identified as causing IEI to 485 (Fig. 1A, B; see all Tables and Supplementary Table 1).

## Novel Inborn Errors of Immunity

Novel gene defects have been found for most categories of IEI, including novel causes of:

- Combined immunodeficiencies (*LCP2* (SLP76) [12], *PAX1* [13, 14], *ITPKB* [15]; *SASH3* [16, 17], *MAN2B2* [18], *COPG1* [19], *IKZF2* [20–23], *CHUK* [24], *IKZF3* [25, 26], *CRACR2A* [27], *CD28* [28]) (Table 1; Supplementary Table 1);
- Combined immunodeficiencies with syndromic features (*MCM10* [29, 30], *IL6ST* [31–33], *DIAPH1* [34]) (Table 2; Supplementary Table 1);
- B cell deficiencies, agammaglobulinemia, or hypogammaglobulinemia (*FNIP1* [35, 36], *SP1I* [37]*, PIK3CG* [38, 39], *POU2AF1* [40], *CTNNBL1* [41], *TNSRSF13* [42]) (Table 3; Supplementary Table 1);
- Immune dysregulation (*RHOG* [43], *SOCS1* [44–46], *PDCD1* [47], *ELF4* [48, 49], *TET2* [50], *CEBPE* [51], *IKZF1* GOF [52]) (Table 4; Supplementary Table 1)
- neutropenia *CXCR2* [53, 54] (Table 5, Supplementary Table 1)
- innate immune defects resulting in susceptibility to mycobacterial/bacterial (*TBX21* [55, 56], *IFNG* [57], *TLR8* [58, 59]), viral (*NOS2* [60], *SNORA31* [61], *ATG4A, MAP1LC3B2* [62], *ZNFX1* [63–65], *TLR7* [66–68]), and/or fungal infections (*MAPK8* [69]) (Table 6; Supplementary Table 1);
- Autoimmune/autoinflammatory disorders (*TMEM173* [70], *LSM11, RNU7-1* [71], *CDC42* [72–78], *STAT2* [79, 80], *ATAD3A* [81], AR *TBK1* [82], *C2orf69* [83, 84], *RIPK1* [85, 86], *NCKAP1L* [87–89], *SYK* [90], *HCK1* [91], *IKBKG* [92–94]); *PSMB9* [95, 96]; and somatic variants in *UBA1* [97]) (Table 7, 10, Supplementary Table 1);
- Bone marrow failure (*MECOM1*) [98, 99] (Table 9; Supplementary Table 1); and
- Phenocopies of IEI (somatic variants in *TLR8* [58], autoAbs against type 1 IFNs [100–104]) (Table 10; Supplementary Table 1).

**Table 1:**
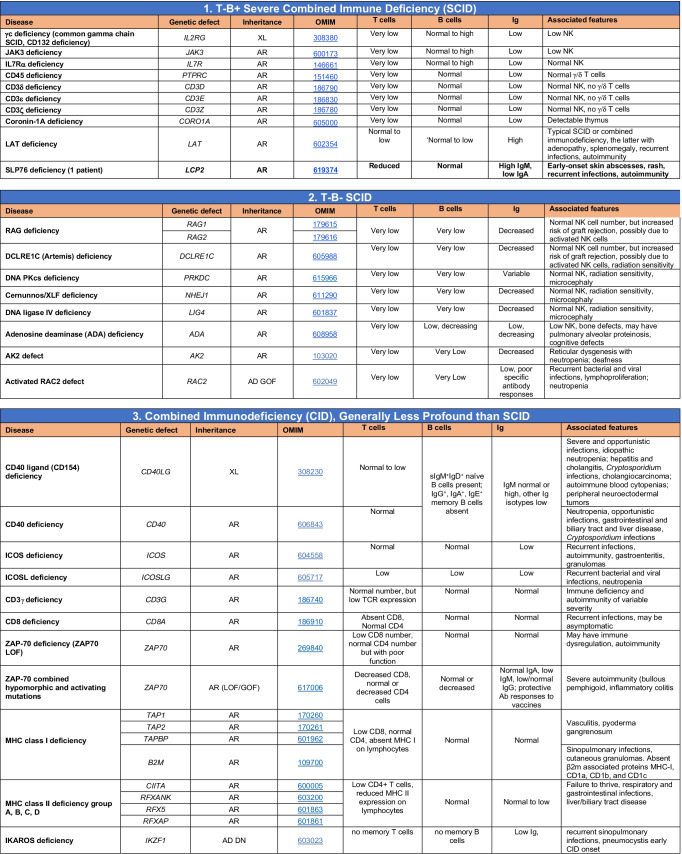

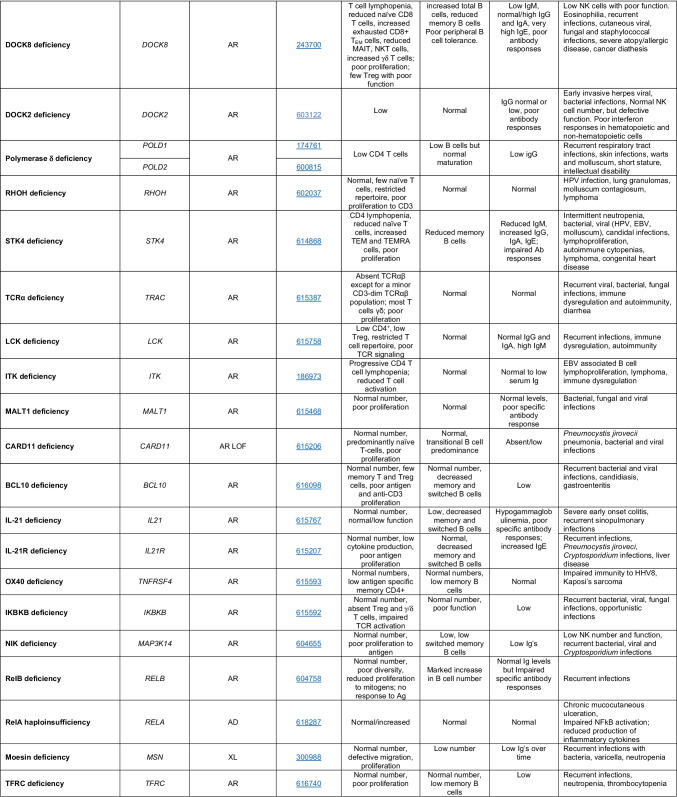

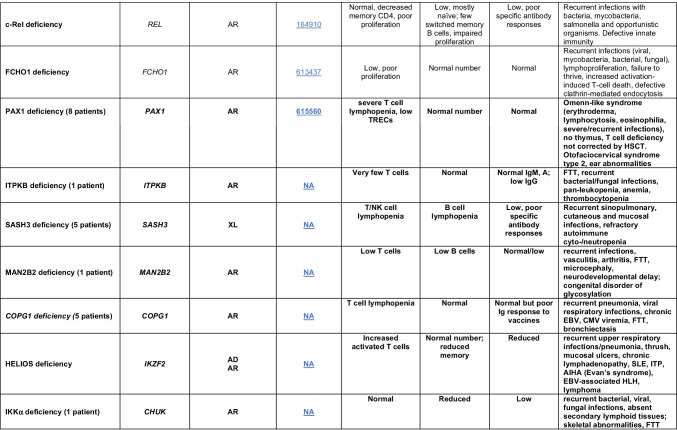
Immunodeficiencies affecting cellular and humoral immunity

SCID/CID spectrum: Infants with SCID who have maternal T cell engraftment may have T cells in normal numbers that do not function normally; these cells may cause autoimmune cytopenias or graft versus host disease. Hypomorphic mutations in several of the genes that cause SCID may result in Omenn syndrome (OS), or “leaky” SCID, or still less profound combined immunodeficiency (CID) phenotypes. Both OS and leaky SCID can be associated with >300 autologous T cells/uL of peripheral blood and reduced, rather than absent, proliferative responses when compared with typical SCID caused by null mutations. A spectrum of clinical findings including typical SCID, OS, leaky SCID, CID, granulomas with T lymphopenia, autoimmunity and CD4 T lymphopenia can be found in an allelic series of *RAG1/2* and other SCID-associated genes. There can be clinical overlap between some genes listed here and those listed in Table 7

Total number of mutant genes: 66. New inborn errors of immunity: 8 (*SLP76* [12], *PAX1* [13, 14], *ITPKB* [15]; *SASH3* [16, 17], *MAN2B2* [18], *COPG1* [19], *IKZF2* [20–23], *CHUK* [24])

*SCID* severe combined immunodeficiency, *CID* combined immunodeficiency, *EBV* Epstein-Barr virus, *MHC* major histocompatibility complex, *HPV* human papillomavirus, *Treg* T regulatory cell, *XL* X-linked inheritance, *AR* autosomal recessive inheritance, *AD* autosomal dominant inheritance, *LOF* loss-of-function, *GOF* gain-of-function, *FTT* failure to thrive

**Table 2:**
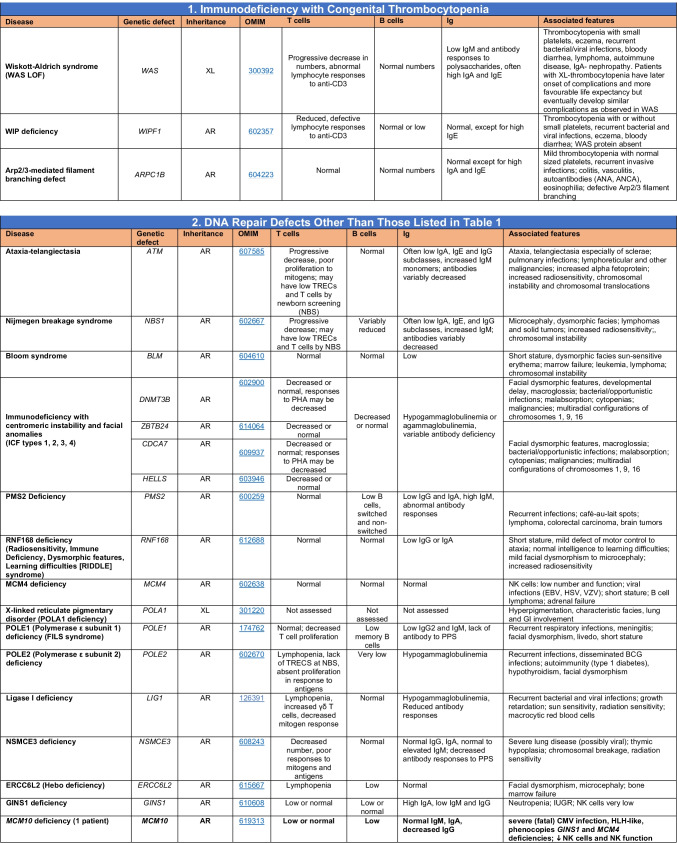

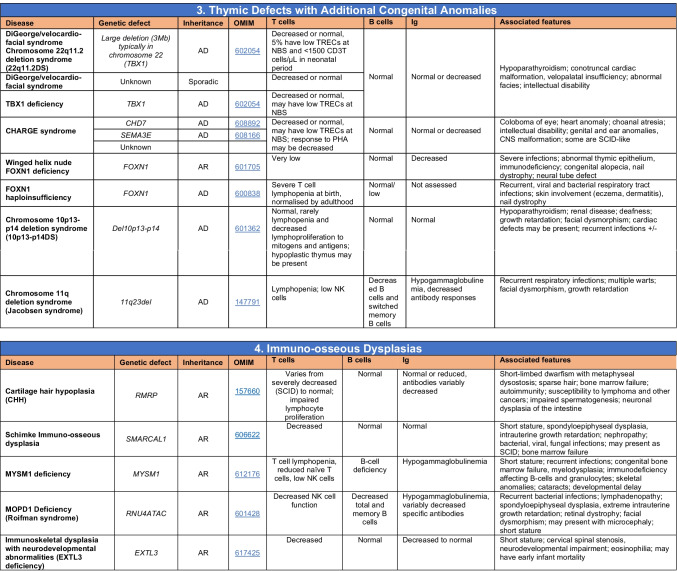

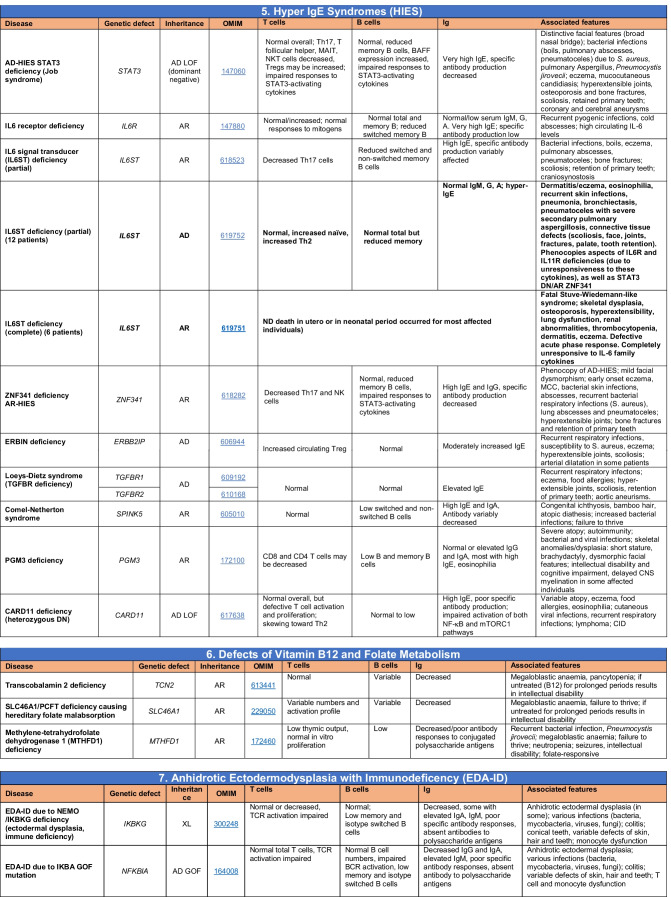

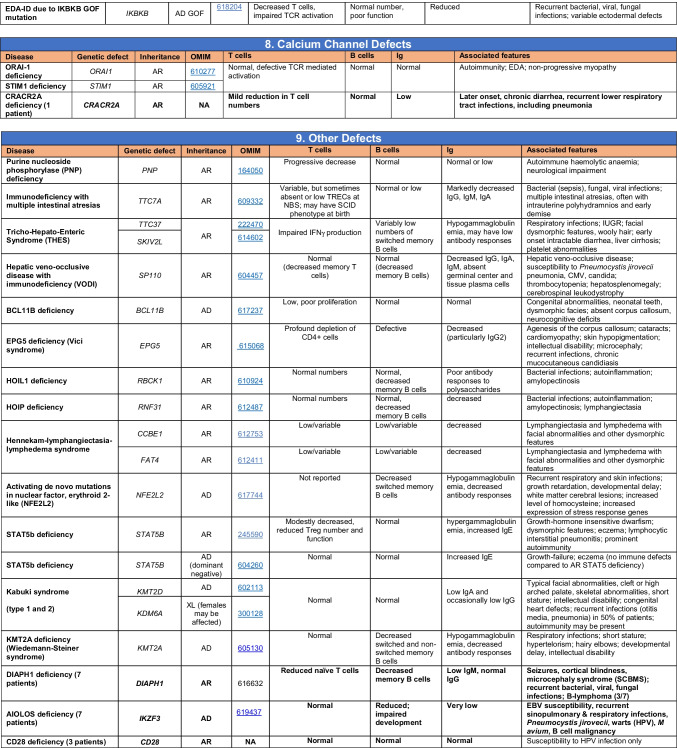
Combined immunodeficiencies with associated or syndromic features

Total number of mutant genes in Table 2: 69. New inborn errors of immunity: 7 (*MCM10* [29, 30], AR and AD *IL6ST* [31–33], *CRACR2A* [27], *DIAPH1* [34], *IKZF3* [25, 26], *CD28* [28]). Unknown cause of DiGeorge syndrome, unknown cause of CHARGE syndrome, unknown gene(s) within 10p13-14 deletion responsible for phenotype

*EDA* ectodermal dysplasia anhidrotic, *HSV* herpes simplex virus, *VZV* varicella zoster virus, *BCG Bacillus* Calmette-Guerin, *NBS* newborn screen, *TREC* T cell receptor excision circle (biomarker for low T cells used in NBS), *IUGR* intrauterine growth retardation

**Table 3:**
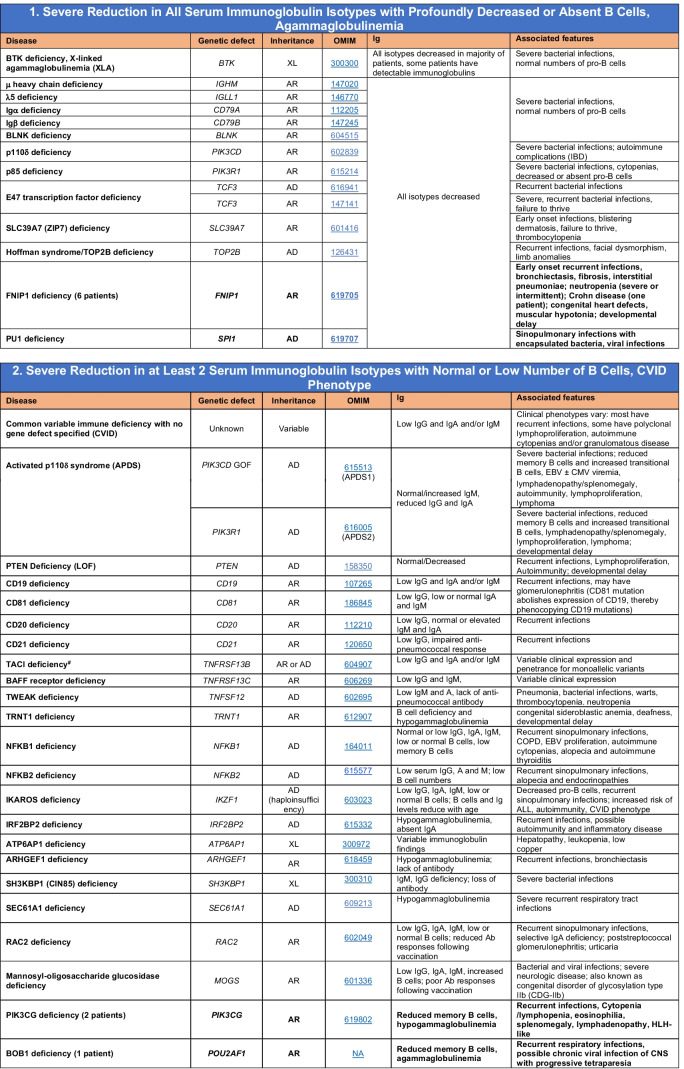

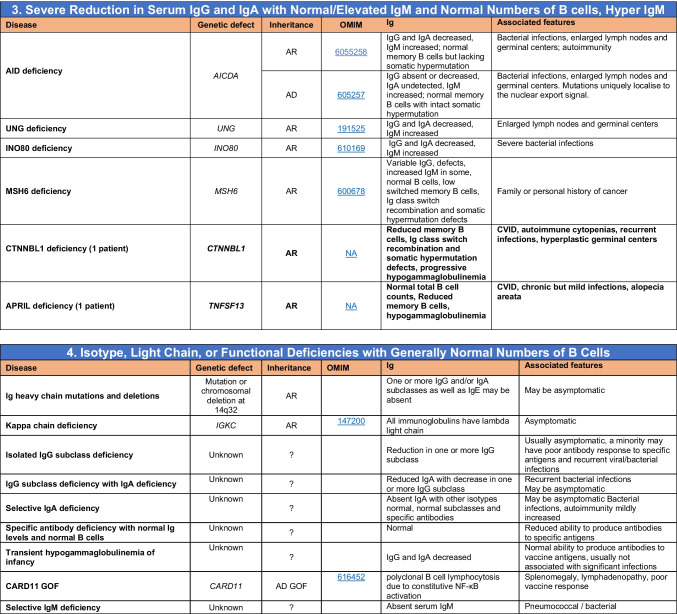
Predominantly antibody deficiencies

Common variable immunodeficiency disorders (CVID) include several clinical and laboratory phenotypes that may be caused by distinct genetic and/or environmental factors. Some patients with CVID and no known genetic defect have markedly reduced numbers of B cells as well as hypogammaglobulinemia. Identification of causal variants can assist in defining treatment. In addition to monogenic causes on this table, a small minority of patients with XLP (Table 4), WHIM syndrome (Table 6), ICF (Table 2), VODI (Table 2), thymoma with immunodeficiency (Good syndrome) or myelodysplasia are first seen by an immunologist because of recurrent infections, hypogammaglobulinemia and normal or reduced numbers of B cells

Total number of mutant genes in Table 3: 45. New inborn errors of immunity: 6 (*FNIP1* [35, 36], *SP1I* [37]*, PIK3CG* [38, 39], *POU2AF1* [40], *CTNNBL1* [41], *TNSRSF13* [42])

*EBV* Epstein-Barr virus, *COPD* chronic obstructive pulmonary disease

^#^Heterozygous variants in *TNFRSF13B* have been detected in healthy individuals, thus such variants are likely to be disease-modifying rather than disease-causing

**Table 4:**
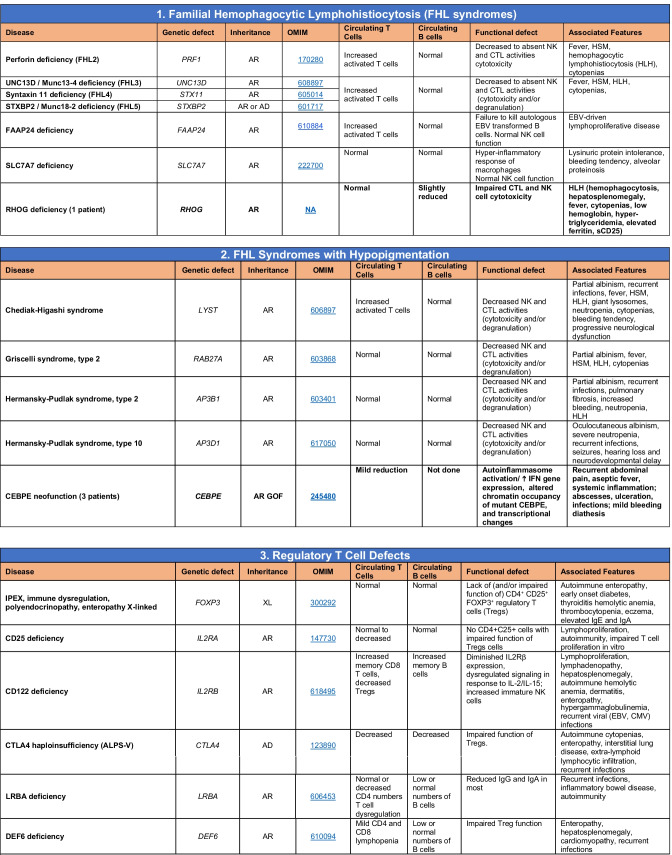

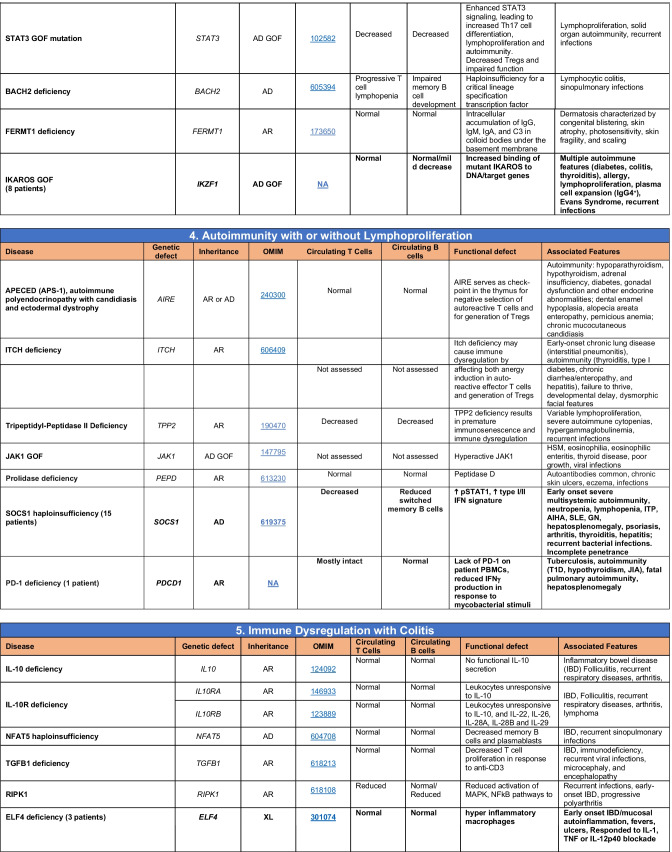

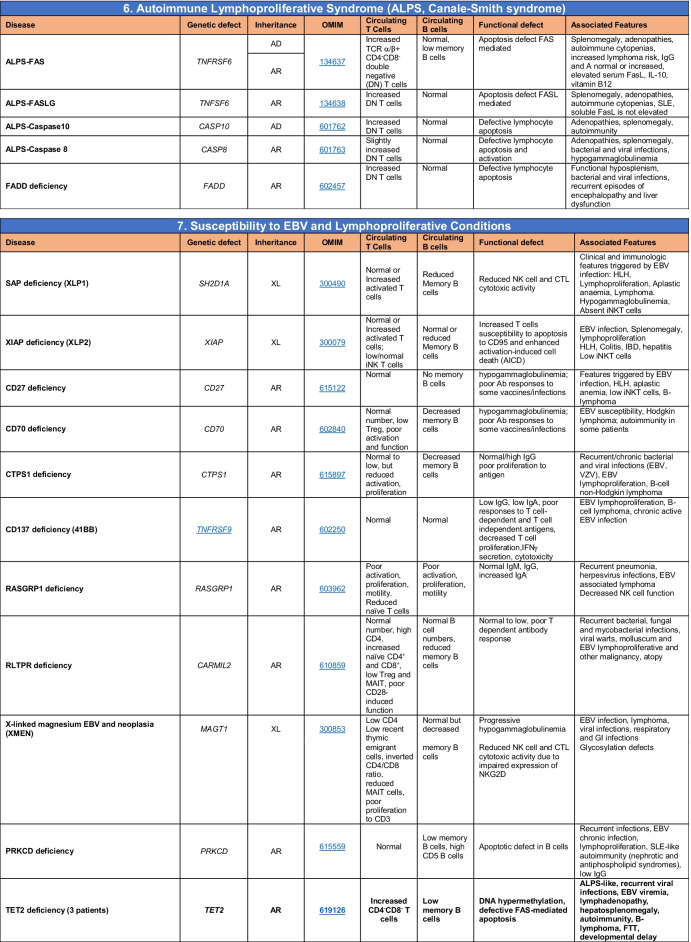
Diseases of immune dysregulation

Total number of mutant genes in Table IV: 52. New inborn errors of immunity: 7 (*RHOG* [43], *CEBPE* [51], AD GOF *IKZF1* [52], *SOCS1* [44–46], *PDCD1* [47], *ELF4* [48], *TET2* [50])

*FHL* familial hemophagocytic lymphohistiocytosis, *HLH* hemophagocytic lymphohistiocytosis, *HSM* hepatosplenomegaly, *DN* double-negative, *SLE* systemic lupus erythematous, *IBD* inflammatory bowel disease

**Table 5:**
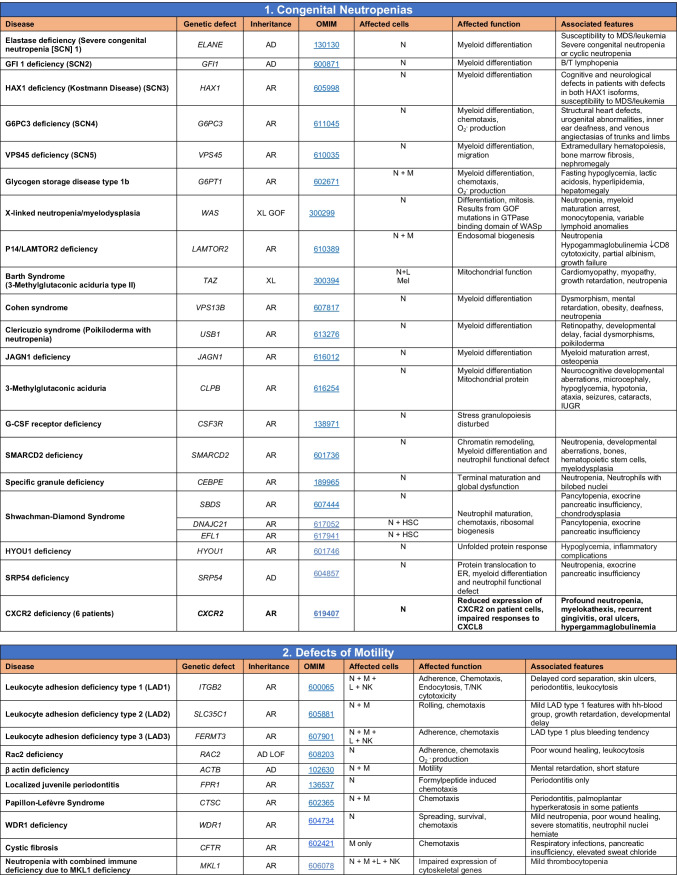

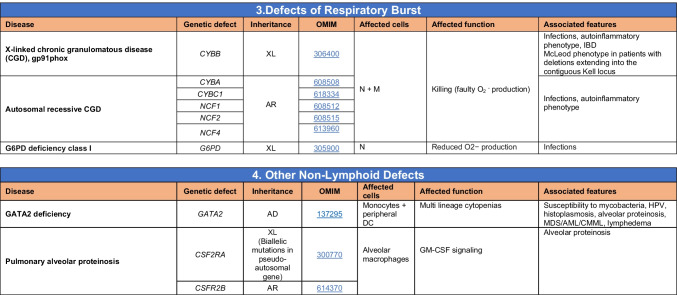
Congenital defects of phagocyte number or function

Total number of mutant genes in Table 5: 42. New inborn errors of immunity: 1 (*CXCR2* [53, 54]). Removed: Cyclic neutropenia was merged with elastase deficiency

*MDS* myelodysplastic syndrome, *IUGR* intrauterine growth retardation, *LAD* leukocyte adhesion deficiency, *AML* acute myelogenous leukemia, *CMML* chronic myelomonocytic leukemia, *N* neutrophil, *M* monocyte, *MEL* melanocyte, *L* lymphocyte, *NK* natural killer

**Table 6:**
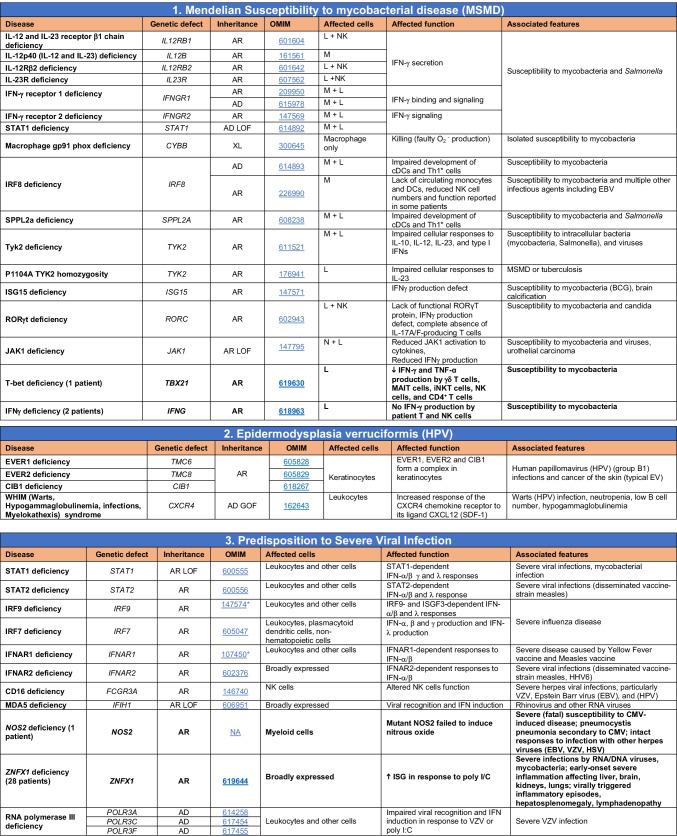

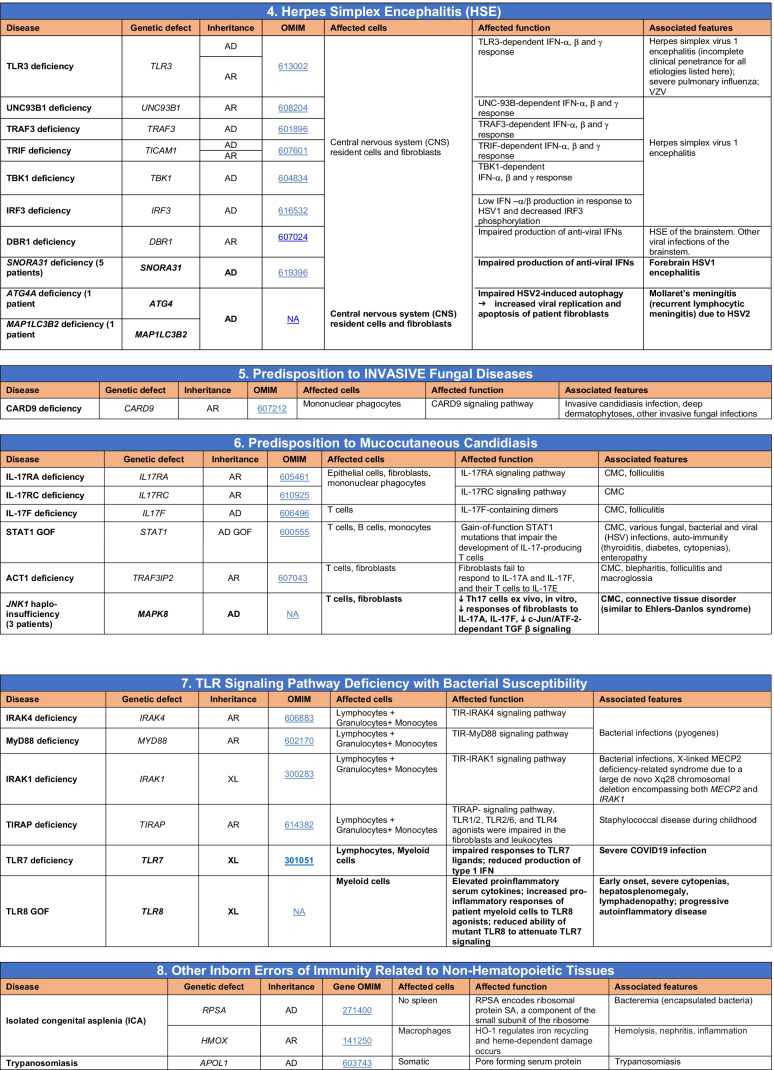

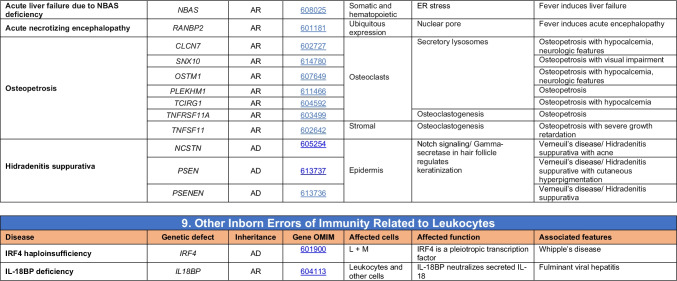
Defects in intrinsic and innate immunity

Total number of mutant genes in Table 6: 74. New inborn errors of immunity: 10 (*TBX21* [55], *IFNG* [57], *NOS2* [60], *ZNFX1* [63–65], *SNORA31* [61], *ATG4A, MAP1LC3B2* [62], *MAPK8* [69], *TLR7* [66–68], *TLR8* [58, 59])

*NF-κB* nuclear factor kappa B, *TIR* Toll and interleukin 1 receptor, *IFN* interferon, *TLR* Toll-like receptor, *MDC* myeloid dendritic cell, *CNS* central nervous system, *CMC* chronic mucocutaneous candidiasis, *HPV* human papillomavirus, *VZV* varicella zoster virus, *EBV* Epstein-Barr virus

**Table 7:**
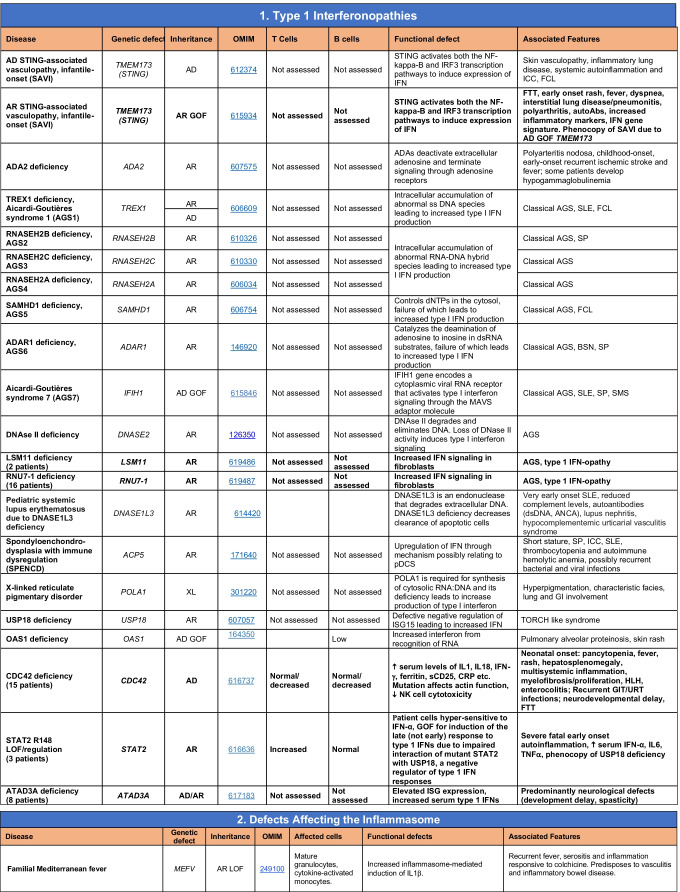

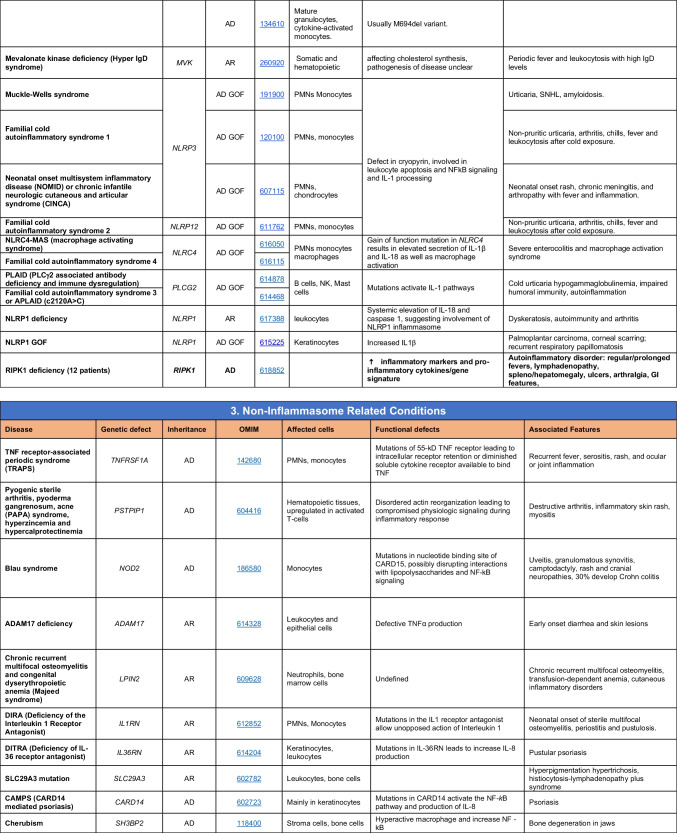

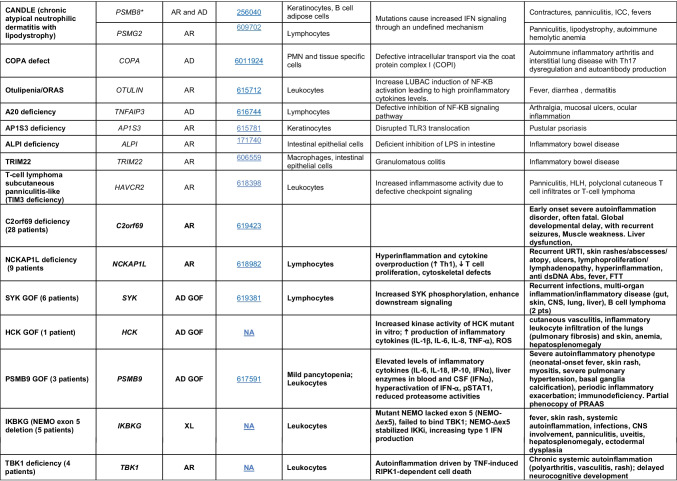
Autoinflammatory disorders

Total number of disorders in Table 7: 56. New inborn errors of immunity: 14 (AR GOF *TMEM173* [70], *LSM11, RNU7-1* [71], *CDC42* [72–78], *STAT2* [79, 80], *ATAD3A* [81], *C2orf69* [83, 84], *RIPK1* [85, 86], *NCKAP1L* [87–89], *SYK* [90], *HCK1* [91], *PSMB9* [95, 96], *IKBKG* NEMO-Δex5, AR *TBK1* [82])

*IFN* interferon, *HSM* hepatosplenomegaly, *CSF* cerebrospinal fluid, *SLE* systemic lupus erythematosus, *TORCH* toxoplasmosis, other, rubella, cytomegalovirus, and herpes infections, *SNHL* sensorineural hearing loss, *AGS* Aicardi-Goutières syndrome, *BSN* bilateral striatal necrosis, *FCL* familial chilblain lupus, *ICC* intracranial calcification, *IFN* interferon type I, *pDCs* plasmacytoid dendritic cells, *SP* spastic paraparesis, *SMS* Singleton-Merten syndrome, *ss* single-stranded DNA

*Variants in *PSMB4*, *PSMB9*, *PSMA3*, and *POMP* have been proposed to cause a similar CANDLE phenotype in compound heterozygous monogenic (*PSMB4*), digenic (*PSMA3/PSMB8*, *PSMB9/PSMB4*, *PSMB4/PSMB8*) and AD monogenic (*POMP*) models [115]

**Table 8:**
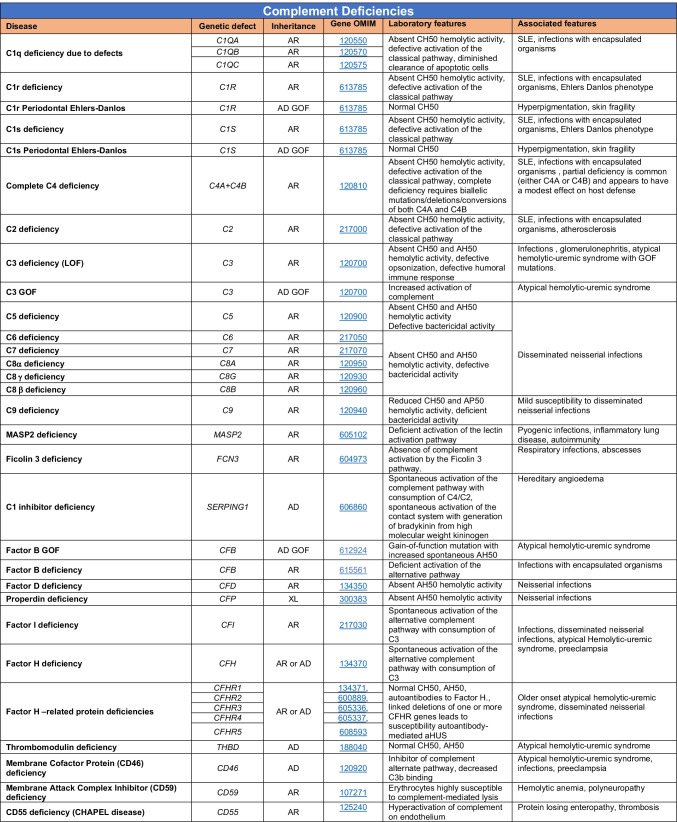
Complement deficiencies

Total number of mutant genes in Table 8: 36. New disorders: Nil

*MAC* membrane attack complex, *SLE* systemic lupus erythematosus

**Table 9:**
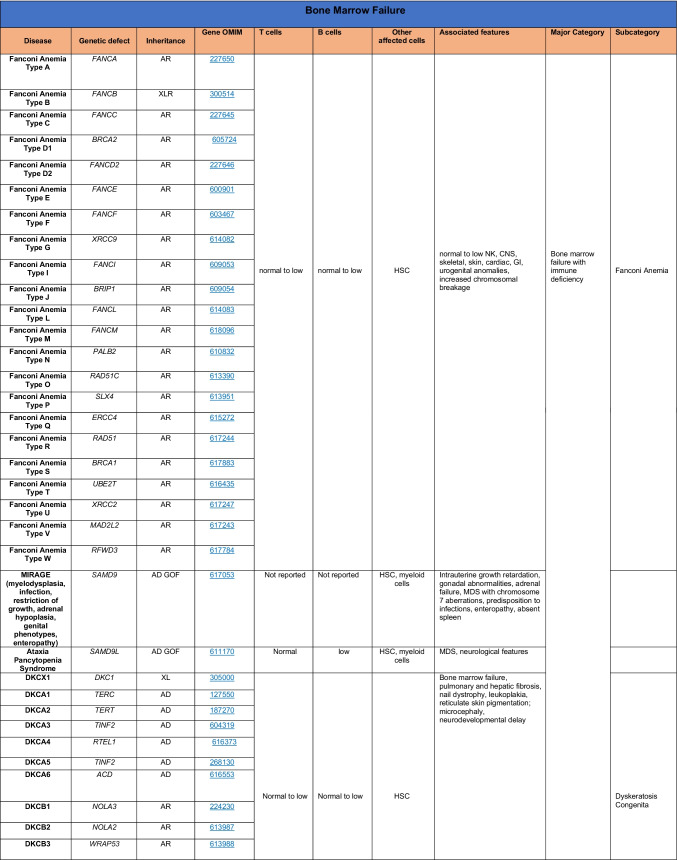

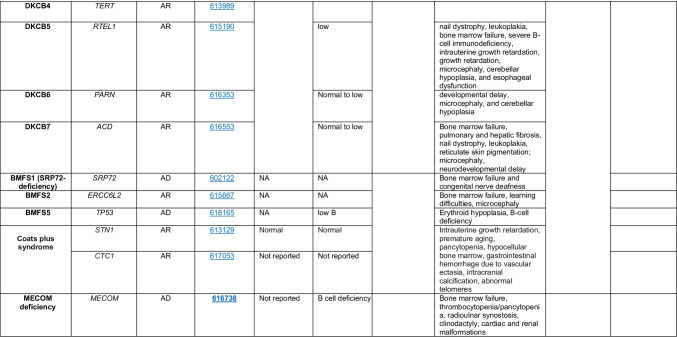
Bone marrow failure

Total number of mutant genes in Table 9: 44. New Inborn errors of immunity: 1 (*MECOM1*) [98, 99])

*HSC* hematopoietic stem cell, *NK* natural killer, *CNS* central nervous system, *GI* gastrointestinal, *MDS* myelodysplastic syndrome, *DKCX* X-inked dyskeratosis congenital, *DKCA* autosomal dominant dyskeratosis congenita, *DKCB* autosomal recessive dyskeratosis congenita, *BMFS* bone marrow failure syndrome

**Table 10:**
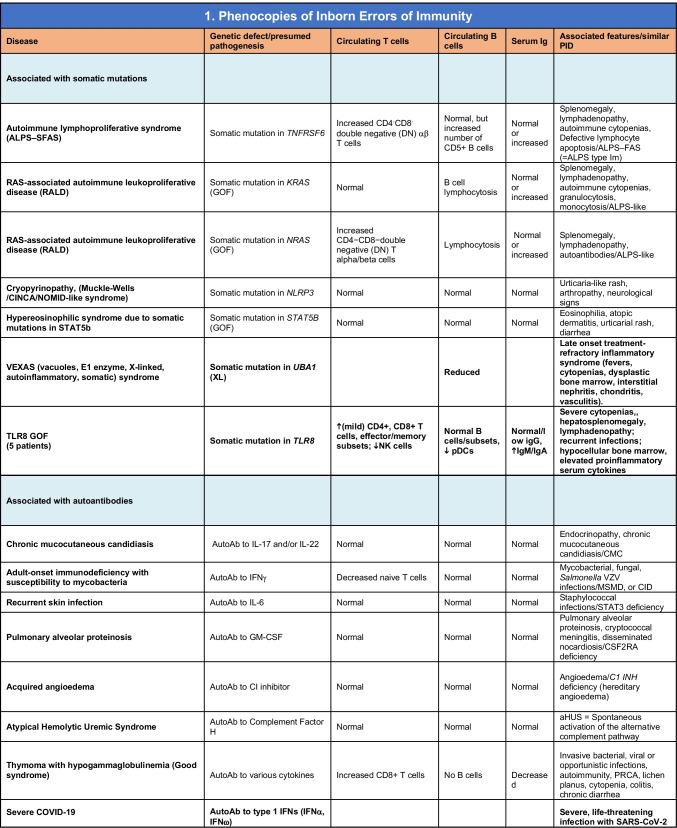
Phenocopies of inborn errors of immunity

Total number of conditions for Table 10: 15 (7 due to somatic mutations; 8 due to autoAbs). New phenocopies: 3 (somatic variants in *UBA1* [97], *TLR8* [58]; autoAbs against type 1 IFNs [100–104])

*aHUS* atypical hemolytic uremic syndrome, *XL* X-linked inheritance, *AR* autosomal recessive inheritance, *AD* autosomal dominant inheritance, *LOF* loss-of-function, *GOF* gain-of-function, *PRCA* pure red cell aplasia

## Novel IEI Phenocopy Known IEI, Confirming Critical Pathways for Immune Function

Some of these novel genetic findings link common clinical phenotypes that converge on a shared pathway. Examples in this update include:

- SLP76, encoded by *LCP2*, is part of the TCR signalosome, interacting with or being downstream of ZAP70, LCK, LAT and ITK [105]. Thus, the phenotype of AR SLP76 deficiency overlaps substantially with that of individuals with mutations in these genes [12].
- MCM10 is a component of the DNA replication machinery of mammalian cells and forms part of multimeric/multiprotein “replisome” complexes [106]. Thus, bi-allelic mutations in *MCM10* result in a clinical phenocopy of AR *MCM4* or *GINS1* variants [29, 30], which also encode key proteins involved in DNA replication [106].
- The non-redundant role of IFNγ-mediated immunity in protection against mycobacterial infection was established by identifying individuals with mutations in not only *IFNG* itself [57], but also *TBX21* [55], the transcription factor that regulates IFNγ, who develop Mendelian susceptibility to mycobacterial disease. T-bet deficiency also resulted in upper airway inflammation and Th2 dysregulation [56], further highlighting immune regulation mediated by opposing functions of transcription factors in T cells with distinct fates (Th1 vs Th2).
- Individuals with complete gp130-deficiency due to bi-allelic mutations of *IL6ST* [33], or dominant negative heterozygous variants of *IL6ST* [31], present with eczema, hyper-IgE, and eosinophilia, similar to individuals with AD hyper-IgE syndrome due to dominant negative mutations in *STAT3* or AR mutation in *ZNF341* [107]. These findings from the different genotypes indicate a key role for IL-6 signaling, via STAT3/ZNF341, in regulating hyper-IgE and atopy.
- Store-operated calcium entry via Ca^2+^-release activated Ca^2+^ channels (CRAC) enable transfer of Ca^2+^ across cell membranes following activation of surface receptors, thereby eliciting Ca^2+^ flux and initiation of key intracellular signals [108]. Bi-allelic LOF variants in *STIM1* or *ORA1* disrupt Ca^2+^ flux, thereby impairing lymphocyte activation following engagement of antigen receptors, resulting in combined immunodeficiencies [108]. The first report of an individual with compound heterozygous inactivating variants in *CRACR2A* provides further insight into the importance of Ca^2+^-dependent signaling in immune cells [27].
- The IKAROS family of proteins — IKAROS, AIOLOS, and HELIOS — interacts with one another as homodimers, heterodimers, or heterotrimers to regulate immune cell development and function [109]. While variants in *IKZF1* encoding IKAROS have been previously reported [5, 109], individuals have now been identified with pathogenic variants in *IKZF2* (HELIOS) [20–23] and *IKZF3* (AIOLOS) [25, 26], as well as GOF variants in *IKZF1* [52]. While these genotypes present with some distinct clinical phenotypes, there is also substantial overlap, such as B cell deficiency, hypo- or agammaglobulinemia, recurrent infections, and predisposition to B cell malignancy.

## One Gene, Several Phenotypes

The discovery of novel IEI continues to demonstrate that distinct types of variants (GOF, LOF, mono-allelic, bi-allelic, exon splicing) in the same gene can cause disparate clinical conditions. This update includes AR and AD forms of *IKZF2* (HELIOS) [20–23] and *IL6ST* [31–33] deficiency, as well as AD *RIPK1* LOF [85, 86], AR GOF *TMEM173/STING* [70], AR LOF *TBK1* [82], and mono-allelic *IKZF1* GOF [52] variants which complement previous reports of AR RIPK1 deficiency, AD GOF *TMEM173/STING*, AD *TBK1* deficiency, and mono-allelic *IKZF1* inactivating variants, respectively [5]. AR GOF variants in *CEBPE* also represent a novel IEI [51]. Notably, these variants resulted in neomorphic function of the C/EBPε transcription factor, causing dysregulated expression of >400 genes, ~15–20% of which are not normally targeted by C/EBPε [51]. This may represent the prototype for neomorphic variants causing IEI.

Intriguingly, specific variants in *STAT2* or *IKBKG* — which are already well-known to cause IEIs — have recently been reported that cause very distinct phenotypes from those previously associated with pathogenic variants in these genes. STAT2 plays a ying/yang role in type 1 IFN signalling. Thus, it is responsible for not only inducing, but also restraining, responses elicited via IFNαR1/2 complexes [110]. This regulatory role of STAT2 is mediated by binding to and recruiting USP18 to IFNαR2, which then prevents further recruitment of JAKs to type 1 IFN receptors, thereby attenuating IFNα signalling [110]. Bi-allelic variants in *STAT2* that specifically affect amino acid R148 (STAT2^R148Q/W^) have now been reported [79, 80]. These STAT2^R148Q/W^ variants are LOF for binding to USP18 [79, 80, 110]. Consequently, STAT2^R148Q/W^ prevents USP18-mediated restraint of type 1 IFN signalling. It is important to appreciate that while STAT2^R148Q/W^ is not intrinsically GOF, the net outcome of loss of STAT2-mediated regulation of type 1 IFN signalling is reminiscent of other Mendelian IFN-opathies. Indeed, STAT2^R148Q/W^ is a phenocopy of USP18 deficiency [110], which is clearly distinct from severe susceptibility to some live attenuated viral vaccines and viral infections typical of individuals with null/nonsense mutations in *STAT2* [110]. Lastly, unique variants in *IKBKG* that result in deletion of exon 5 were found to cause an autoinflammatory disease which is also very different from ectodermal dysplasia and immunodeficiency that is typically associated with hypomorphic *IKBKG* variants that impair NEMO expression and/or function [92–94].

Somatic/mosaic disease-causing mutations in *TLR8* [58] and *UBA1* [97] have also been identified, even though the pathogenic alleles were detected in only 5–30% of most blood cells (*TLR8*) [58] or 50–85% of myeloid cells but not in lymphocytes of fibroblasts (*UBA1*) [97]. These findings are an important reminder to consider the nature of genetic variants identified from unbiased next-generation sequencing, recognizing multiple mechanisms of pathogenicity for the same gene. This is highlighted by at least 40 genes having multiple entries in the current update to reflect these distinct modes of disease pathogenesis (Supplementary Table). This also emphasizes the crucial need to undertake in-depth in vitro functional validation of any variant considered to be potentially pathogenic. Alternatively, it signifies the difficulty in excluding a candidate pathogenic variant without functional testing. It also underscores the need to consider variants detected at low allelic frequencies that may represent somatic/mosaic, rather than germline, variants. These findings also predict that somatic variants in key immune genes will be frequently discovered as causes of novel IEI in the not-too distant future [111].

## IEI Define Specific Roles for Known Genes and Reveal Immune-Specific Functions of Novel Genes

One of most profound outcomes of discovering the genetic cause of an IEI is the ability to ascribe unequivocally non-redundant, as well as redundant, functions to a specific gene in human immunity. Classic examples of this are the fundamental requirement for *IL2RG* in humans for the development of T and NK cells, but not B cells, and the essential role of STAT3 for CD4^+^ T cell differentiation into Th17 cells and subsequent host defense against fungal infections, but not for the generation of most other CD4^+^ T cell effector populations [112]. Findings included in this update confirm data from mice on the importance of *FNIP1* and *SPI1* (encoding PU.1) during human B cell development [35–37] and the fundamental regulatory role of PD-1 (encoded by *PDCD1*) in human immune function [47]. However, and perhaps counter to all expectations and immunology dogma relating to T cell co-stimulation, CD28 is required for host defense against HPV but is largely redundant in the face of other infectious pathogens [28]. Who would have thought!

The latest IEI have also revealed critical roles for genes not previously strongly associated with immune regulation and/or host defense. For instance, we have now learned that:

- The SH3-domain containing protein SASH3 contributes to B and T cell developments [16, 17].
- *ZNFX1*, a member of an RNA helicase superfamily, plays a dual role in human immunity, including in innate immune responses against viruses, bacteria, mycobacteria, and fungi, as well as in restraining type 1 IFN-mediated inflammation [63–65].
- The small nucleolar RNA *SNORA31* plays a critical role in CNS-intrinsic immunity against HSV-2 infection, likely via production of type 1 IFN, yet the exact mechanism remains unknown [61].
- The hitherto uncharacterized protein-coding gene *C2orf69* has a multitude of roles across numerous biological systems, including regulating autoinflammation [83, 84].

The discovery of these novel IEIs provides opportunities to further extend our understanding of human immunity and immune regulation.

## SARS-CoV2 and Inborn Errors of Immunity

The emergence of novel pathogens poses potential health risks to the general population due to the lack of substantial pre-existing immune memory. More critically though, individuals with specific germline genetic variants — causing known and unknown IEIs — may be at greater risk of experiencing more severe disease following infection than the general population. The COVID-19 pandemic has indeed revealed genes and pathways essential for anti-SARS-CoV2 immunity. Genomic studies discovered that ~2–3% of cases of severe life-threatening SARS-CoV2 infection resulted from germline LOF/LOE variants in the type 1 IFN signaling pathway: *TLR3, UNC93B1, TICAM1, TBK1, IRF3, IRF7, IFNAR1*, and *IFNAR2* [113]. These findings are reminiscent of earlier studies that identified variants in these genes in individuals susceptible to life-threatening infections with other viruses, including influenza virus, HSV-1, and live viral vaccines [114]. Hemizygous deleterious variants have also been identified in *TLR7* in ~1% of males who developed severe/fatal COVID-19 [66–68]. Thus, X-linked TLR7 deficiency represents a novel IEI predisposing to severe COVID-19.

The importance of type 1 IFN in anti-SARS-CoV2 immunity was also realized by the finding that ~10–20% of patients with severe COVID-19 have high levels of neutralizing serum autoantibodies (autoAbs) against type 1 IFNs; these were not detected in asymptomatic infected individuals [100–104]. Collectively, these studies defined a non-redundant role for type 1 IFNs in host defense against SARS-CoV2 infection and established that autoAbs against type 1 IFN phenocopy an IEI.

## Conclusions

The goals of the IUIS Expert Committee on IEI are to increase awareness, facilitate recognition, promote optimal treatment, and support research in the field of clinical immunology. Since the last IEI update, we have continued to witness the ongoing rapid identification, and molecular, biochemical, and cellular characterization, of genetic variants that cause human diseases by disrupting host defense or immune regulation. The 55 novel gene defects reported here bring to total number of IEI to 485 (Fig. 1A, B), thus underscoring the power of next-generation sequencing technologies and sophisticated functional validation of candidate pathogenic variants to (1) identify novel gene defects underlying human disease, (2) elucidate mechanisms of disease pathogenesis, (3) define non-redundant functions of key genes in human immune cell development, host defense and immune regulation, (4) expand the immunological and clinical phenotypes of IEI, and (5) implement gene-specific therapies. These fundamental discoveries continue to highlight the critical contributions of IEI to our broader understanding of basic, translational, and clinical immunology, as well as molecular medicine. And we will no doubt observe novel insights into basic and clinical immunology with the next wave of novel IEIs.

## Supplementary Information

Below is the link to the electronic supplementary material.

Supplementary file1 (DOCX 350 kb)

Supplementary file2 (XLSX 93 kb)

## Data Availability

Not applicable
