# Supplementary material for: Human Inborn Errors of Immunity: 2022 Update on the Classification from the International Union of Immunological Societies Expert Committee
Source: J Clin Immunol. 2022 Jun 24;42(7):1473–507. doi: 10.1007/s10875-022-01289-3 (PMC9244088; doi:10.1007/s10875-022-01289-3)
Supplement: Supplementary file 1 — Supplementary file1 (DOCX 350 kb) [file 10875_2022_1289_MOESM1_ESM.docx]

**Supplementary Table 1: Newly validated Inborn Errors of Immunity - 2022**

| **Novel Gene Defects Underlying IEI – 2022** | | | | | | | |
| --- | --- | --- | --- | --- | --- | --- | --- |
| **Genetic defect** | **Inheritance/mechanisms** | **T/NK cells** | **B cells** | **Ig levels** | **Clinical features, cellular defects and evidence of variant pathogenicity** | **Table (for classification)^[1, 2]^** | **Refs** |
| ***LCP2*** (SLP76)  (1 patient) | AR (LOF) | • reduced T cells,  • 🡓🡓CD4+, 🡑 CD8+ T cell proportions  • Low naïve, 🡑T_CM_ CD4+ T cells; • CD8+ T cells mostly clonally expanded T_EMRA_  • Low TRECs  • NK normal numbers | • Normal numbers  • 🡓class switched memory and transitional B cells  • 🡑naïve and immature  B cells | • High IgM, low IgA | •  combined immunodeficiency,  • early-onset skin abscesses, rash, recurrent infections, autoimmunity,  • neutrophil and platelet dysfunction  • 🡓 T cell proliferation to PHA, anti-CD3/CD28 stimulation, partially restored by IL-2  • 🡓 NK cell degranulation  • 🡓 actin polymerization  **Validation**  • generation of SLP76-deficient Jurkat T cells; expressing WT or mutant *SLP76* allele, mimicking patient cellular phenotypes;  • partial rescue of some of the functional defects by expression of WT *LCP2/SLP76* | Table 1  Subtable 1 | [3] |
| ***PAX1*** (8 patients, 3 families; 2 papers with overlapping patients) | AR (LOF) | • T^-^B^+^NK^+^ SCID  • Severe T lymphopenia, low TRECs, | • Normal | • normal IgM, low/normal IgG, low IgA, normal/🡑 IgE | • Omenn-like syndrome (erythroderma, lymphocytosis, eosinophilia, 🡓 proliferation to PHA, severe/recurrent infections),  • no thymus, T cell deficiency not corrected by HSCT despite donor chimerism  • also: otofaciocervical syndrome type 2 (OTFCS2)  • ear abnormalities  **Validation**  •  reporter assays established hypomorphic LOF of mutant alleles  • patients-derived iPSCs differentiated to thymic epithelial cells demonstrated abnormalities in thymic epithelial progenitors, consistent with failure of HSCT to reconstitute T cells  • similar to mouse model | Table 1  Subtable 3 | [4, 5] |
| ***ITPKB***  (1 patient) | AR | • T^-^B^+^NK^+^ SCID  • 🡓🡓 T cells, normal NK cells | • Normal | Normal IgM, A; low IgG | • FTT  • recurrent bacterial/fungal infections, • leukopenia/lymphopenia/neutropenia/anemia/ thrombocytopenia. • Fatal  **Validation:** functional validation of variant in transfected cells, as well as using an inhibitor of ITPKB  **Rescue: not** done | Table 1  Subtable 3 | [6] |
| ***SASH3***  (5 patients, unrelated) | XL | • CD4+ T cell, NK cell lymphopenia  • 🡓 naïve CD4 and CD8 T cells | • B cell lymphopenia  • 🡑 BM plasmablasts | • low serum IgG, IgA  • reduced specific Ab responses | • combined immunodeficiency and immune dysregulation  • recurrent sinopulmonary, cutaneous and mucosal infections,  • refractory autoimmune cytopenias neutropenia  • increased levels of pro-inflammatory biomarkers, consistent with immune dysregulation (eczema, auto-immunity)  • 🡓TCR-induced T cell signaling, proliferation/cell cycle progression, 🡑 apoptosis  **Validation:** Lentivirus-mediated transfer of SASH3 cDNA restored protein expression, in vitro proliferation, signaling in patient-derived T cells and Jurkat cells | Table 1  Subtable 3 | [7, 8] |
| ***MAN2B2***  (1 patient) | AR | No thymic shadow,  🡓 T cells (🡓 naïve, 🡑 T_EMRA_), no TRECs  🡓 TCR proliferation | 🡓 total B cells | ~normal IgM, low IgA, 🡑 IgE | CID, recurrent infections, vasculitis, arthritis, FTT, microcephaly, neurodevelopmental delay; congenital disorder of glycosylation  **Validation:** yes  **Rescue:** somewhat | Table 1  Subtable 3 | [9] |
| ***COPG1***  *(*5 patients; 1 family [sibs]) | AR | • T cell lymphopenia, mostly CD4 | • normal slightly reduced | • near-normal levels of serum IgM, G, A  • poor/absent Ig responses to vaccines | • recurrent pneumonia, viral respiratory infections, bronchiectasis, • chronic EBV, CMV viremia  • failure to thrive  impaired T and B cell function in vitro  **Validation:** extensive characterization of impact of mutation on protein function  • Copg1 mutant mice phenocopy defects in B and T cells, develop disease in presence of natural microbial exposure | Table 1  Subtable 3 | [10] |
| ***IKZF2*** (HELIOS)  (AD: 8 patients, 6 families;  AR: 1 patient) | AD  AR | • 🡑 activated/ exhausted T cells  • 🡑 production pro-inflammatory cytokines by T cells  • 🡓 MAIT, Tfh cells  • 🡓 NK cells  • normal Tregs | • normal B cell numbers, but low switched memory  • dysregulated germinal centers | • hypogammaglobulinemia  • low/normal IgG vaccine responses | • combined immunodeficiency  • recurrent upper respiratory infections/pneumonia, thrush,  • mucosal ulcers, chronic lymphadenopathy, SLE, ITP, AIHA (Evan’s syndrome)  • EBV-associated HLH  • lymphoma  **Validation:** variant abolished homodimerization as well as heterodimerization with Helios, Ikaros,  • prevented Helios function, binding to target genes  • similar phenotype as *Ikzf2* homozygous deficient mice | Table 1  Subtable 3 | [11-14] |
| ***CHUK*** (*IKKA*)  (1 patient) | AR | • Normal T, NK cell numbers  • Accumulation of immature NK cells | • B cell lymphopenia | Pan-hypogammaglobulinemia | • Combined immunodeficiency  • recurrent infections (*Staphylococcus aureus*, mucocutaneous candidiasis, epidermodysplasia verruciformis due to HPV)  , absent secondary lymphoid tissues  • lymphocytic infiltration of intestine and liver  • skeletal abnormalities, failure to thrive  • normal T cell proliferation, reduced NK function  **Validation:** Homozygous IKKa^Y580C/Y580C^ mutant mice phenocopy patient findings | Table 1  Subtable 3 | [15] |
| ***MCM10***  (1 patient) | AR (LOF) | • mild lymphopenia  • 🡓 T_CM_, T_EM_ cells  • 🡓🡓🡓 NK cells (🡑 CD56^bright^, nearly absent CD56^dim^ mature NK) | • 🡓 B cells | • slightly 🡓 IgG, normal IgM/A | • severe (fatal) CMV infection  • HLH-like (based on biomarkers, not clinical features  • phenocopies *GINS1* and *MCM4* deficiencies  • 🡓 NK function  **Validation**  •  detailed functional analysis of *MCM10* variant in primary fibroblasts, transfectants,, iPSC, and CRISPR/Cas9-gene edited NK92 human NK cell line  • assessed mutant allele on NK cell development in humanized mice reconstituted with CD34^+^ HSC generated from the patients or healthy donor IPSCs | Table 2  Subtable 2 | [16, 17] |
| ***IL6ST*** *(*gp130, partial)  (12 patients, 8 families) | AD (DN) | • Normal T cell numbers  • 🡑 naïve CD4 and CD8 T cell proportions  • 🡓 T_CM_ CD4+ and CD8+ T cells and T_EM_ CD8 T cells;  • 🡓 MAIT, Tfh cells,  • 🡑 Th2,  • low to normal NK cell counts | • Normal numbers of B cells, low memory B cells | • Normal/low IgG, A,  • normal IgM,  • hyper IgE.  • vaccine IgG normal | • HIES (similar to STAT3 DN/AR ZNF341);  • dermatitis/eczema, eosinophilia, recurrent skin infections, pneumonia, bronchiectasis, pneumatoceles with severe secondary pulmonary aspergillosis, connective tissue defects (scoliosis, face, joints, fractures, palate, tooth retention)  • Phenocopies aspects of IL6R and IL11R deficiencies (due to unresponsiveness to these cytokines)  **Validation**  **•**LOF and DN alleles shown by overexpression in GP130 deficient HEK293T cells;  • impaired GP130/STAT3 signaling (mostly downstream of IL-6) in patient fibroblasts and leukocytes | Table 2  Subtable 5 | [18] |
| ***IL6ST*** *(*gp130*;* complete deficiency);  (6 patients, 4 families) | AR (LOF) | ND (death in utero or in neonatal period occurred for most affected individuals) | | | • Fatal Stuve-Weidemann-like syndrome; skeletal dysplasia, osteoporosis, hyperextensibility, lung dysfunction, renal abnormalities, thrombocytopenia, dermatitis, eczema  • Defective acute phase response  • Complete unresponsiveness to IL-6 family cytokines  **Validation**  **•**complete LOE (for one allele) and LOF for two alleles tested by overexpression in GP130 deficient HEK293T to all cytokines tested of the IL-6 family.  • Effects of variants well-characterized, including a partial rescue of patient amniocytes | Table 2  Subtable 5 | [19, 20] |
| ***CRACR2A***  (1 patient) | AR | • mild CD4 T cell lymphopenia (🡓 naïve CD4 T)  • Normal NK cell numbers | • Normal B cell numbers • 🡓 switched memory B cells | • panhypogamma (🡓🡓IgG, undetectable IgM, IgA)  • intact B cell function in vitro | • later onset CID with chronic diarrhea (19 yrs)  • recurrent lower respiratory tract infections, including pneumonia  • 🡓 T cell activation/proliferation in vitro  **Validation**  **• r**educed expression of CRACR2A protein in patient PBMCs  • reduced Ca flux/JNK activation, cytokine secretion by patient T cells  • *CRACR2A*^-/-^ T cell line recapitulated defects in cytokine production, TCR signaling; rescued by WT but not patient *CRACR2A* alleles  • phenocopies T cell defects in *Cracr2a* ko mice; some overlapping defects with *STIM1*- or *ORA1*-deficiency | Table 2  Subtable 8 | [21] |
| ***DIAPH1***  *(*7 patients, 5 families) | AR | • 🡓 naive T cells,  • 🡓 RTE’s | •  🡑 naïve / trans,  • 🡓 memory | • dysgamma  🡓IgM, normal IgG, variable IgA  • near normal vaccine resp | • seizures, cortical blindness, microcephaly syndrome (SCBMS)  • recurrent bacterial, viral, fungal infections  • B-lymphoma (3/7)  • 🡓 T cell activation/proliferation in vitro,  • impaired adhesion/MTOC repositioning to immune synapse  • defective cytoskeletal organization/mitochondrial dysfunction in SCBMS pathogenesis  **Validation:** • CRISPR ko in control T cells re-capitulated T cell defects in patients | Table 2  Subtable 9 | [22] |
| ***IKZF3*** *(AIOLOS)*  (7 patients, 2 families) | AD (DN) | • Normal numbers of T, NK cells  • 🡑 naïve CD4+ T cells, RTE  • 🡓 memory, Tfh; • 🡓 CD40L induction  •  🡑 %’s CD8 T cells; 🡑 activated T cells | • abnormal B cell development  (🡓 BM B precursors) • 🡑 CD21^low^ B;  • 🡓 memory B cells  • 🡓 response to CD40 | • severe hypogamma | • EBV susceptibility  • recurrent sinopulmonary/respiratory infections  • *Pneumocystis jirovicii*, warts (HPV), *M avium*  • B cell lymphoma (2/3), B-CLL  **Validation:** functional biochemical analysis of the variant in transfectants; mechanism of dominant negative  • mouse model with same variants recapitulated B cell developmental defect, hypogamma and loss of Tfh cells  **Rescue: not done** | Table 2  Subtable 9 | [23, 24] |
| ***CD28***  (3 patients, 1 family) | AR | • Normal numbers of T and myeloid cells  • 🡓 NK cells | • Normal numbers of total B cells and subsets | • Normal serum IgM, G, A; • intact levels of pathogen-spec IgG | • susceptibility to HPV infection only.  **Validation:** lack of protein expression in T cells, transfectants  • defective responses of T cells to CD28 stimulation  • extensive analysis of immune cell function, phenotypes  • CD28-deﬁcient mice susceptible to cutaneous infections with mouse papilloma virus MmuPV1 | Table 2  Subtable 9 | [25] |
| ***FNIP1***  (6 patients; 5 families) | AR (LOF) | • Mild T cell lymphocytosis | • B-cell lymphopenia (absent/low; BM block [few immature B cells]) | Agamma/ hypogamma | • early onset recurrent infections (ENT, pulmonary, gut)  • bronchiectasis, fibrosis, interstitial pneumoniae  • congenital heart defects (eg hypertrophic cardiomyopathy), muscular hypotonia  • variable neutropenia (severe or intermittent)  Crohn disease (one patient)  • developmental delay  •  increased AMPK activity  **Validation**  • almost recapitulates the mouse model [26, 27] | Table 3  Subtable 1 | [28, 29] |
| ***SPI1*** (PU1)  (6 patients, 6 unrelated families) | AD | • normal numbers of T and NK cells, increased % CD8’s | • 🡓🡓B cells, conventional DCs  • B cell development arrested at pro🡒pre–B stage | • undetectable | • PU.1 deficiency  • sinopulmonary infections with encapsulated bacteria  • viral infections in some patients incl paralysis in 1 following live polio vaccine  **Validation:** reduced PU.1 expression in PU1^hi^ cells (Mo, cDC)  • *SPI1* mutations impair PU.1 expression, nuclear localization, target DNA binding | Table 3  Subtable 1 | [30] |
| ***PIK3CG***  (2 patients; 2 unrelated families) | AR (LOF) | • Normal CD4,  • 🡓 Treg,🡓 CD8 | • Normal but 🡓 memory B cells | • Hypogamma, • intact vaccine responses | •  cytopenia/lymphopenia, eosinophilia, lymphadenopathy, splenomegaly,  •  recurrent infections  • HLH-like; 🡑 inflammatory markers  **Validation**  •  🡓 T cell proliferation, activation in vitro  • cellular defects recapitulated in *PIK3CG* targeted Jurkat T cell line and *Pik3cg* ko mice | Table 3  Subtable 2 | [31, 32] |
| ***POU2AF1*** (BOB1)  (1 patient) | AR | • Normal T cell numbers  • 🡓 cTfh cells | • Normal B cell numbers  • 🡓 switched memory B cells  • altered B cell phenotype | • agamma | • recurrent respiratory infections  • possible chronic viral infection of CNS with progressive tetraparesia  **Validation:** lack of protein expression in EBV-B LCLs and transfected cell lines  • impaired B cell responses in vitro  • rescue of aberrant B cell phenotype and impaired Ig secretion in vitro by lentiviral expression of WT BOB1 in mutant B cells  • phenotype overlap in B cells from patient and *Pou2af1*-deficient mice | Table 3.  Subtable 2 | [33] |
| ***CTNNBL1***  (1 patient) | AR (LOF) | • 🡓 T cells  • 🡓 Treg cells | • Reduced memory B cells  • impaired CSR, SHM  • 🡑 CD21^lo^ autoreactive B cells | • Progressive severe hypogamma | • CVID, autoimmune cytopenias, hypogamma, recurrent infections, hyperplastic germinal centers  **Validation**  • detailed functional analysis of *CTNNBL1* variant in EBV B lines, and engineered RAMOS cell lines;  • mutant allele reduces the binding of CTNNBL1 to AID, with impaired nuclear translocation of AID;  • defective SHM rescued in mutant Ramos cells by WT *CTNNBL1* | Table 3  Subtable 3 | [34] |
| ***TNFSF13*** *(*APRIL);  (1 patient) | AR (LOF) | • Normal T/NK cells | • Normal total B cell counts,  • 🡑 IgM+/🡓 switched memory B cells,  • 🡓 blood plasmablasts | • hypogamma | CVID, chronic but mild infections  , Alopecia areata  **Validation**  • LOE and LOF allele,  • functional analysis of the variant in PBMCs and overexpression;  • impaired function of iPSC-moDC in promoting B cell differentiation could be rescued with exogenous APRIL | Table 3  Subtable 3 | [35] |
| ***RHOG***  (1 patient) | AR | • normal number of T cells  • 🡓 total CD4^+^ and CD8^+^ T cells  • 🡓/absent effector/memory CD8+ T cells  • 🡓🡓 NK cells | •mild 🡓 total B cells | • 🡑 IgM, IgG, normal IgA, IgE | • clinical features of HLH (hemophagocytosis, hepatosplenomegaly, fever, cytopenias, low hemoglobin, hyper-triglyceridemia, elevated ferritin, sCD25)  **Validation:** lack of RhoG expression in patient T cells and fibroblasts  • impaired CTL and NK cell cytotoxicity due to role of RhoG in regulating exocytosis of cytotoxic granules  • targeting RHOG in NK cell lines reduced cytotoxic activity | Table 4  Subtable 1 | [36] |
| ***SOCS1***  (15 patients; 10 families) | AD (by haploinsufficiency) | • 🡓CD4, CD8 T cells | • Predominantly naïve B cells;  • 🡓 switched memory,  • 🡑 CD21^low^ B (autoreactive cells) | • 🡓IgM, G, A  but protective specific Abs; • ANA’s, autoAbs  • 🡑 serum BAFF | • Early onset of severe multisystemic autoimmunity (flared in context of infection-induced inflammation), ITP, AIHA, SLE, GN, hepatosplenomegaly, psoriasis, arthritis, thyroiditis, hepatitis  • Evan’s syndrome (AIHA and ITP)  • recurrent bacterial infections,   •1 patient developed COVID19/MIS-C  • neutropenia, lymphopenia  • incomplete penetrance  **Validation**  • 🡑 pSTAT1, 🡑 type I/II IFN signature  • reporter assays confirmed impaired inhibition of mutant SOCS  • Jakinib effective in vitro and in vivo. | Table 4  Subtable 4 | [37-39] |
| ***PDCD1***  (1 patient; 1 deceased sibling [not genotyped but similar disease history]) | AR | • mild lymphopenia but mostly intact  • 🡑 CD4-CD8- DN T cells  • 🡓 Vδ2+ γδ T, MAIT and CD56^hi^ NK cells | • normal | • high serum IgG, IgA; normal IgM, IgE  • anti-insulin autoantibody, | • tuberculosis, autoimmunity (T1D, hypothyroidism and JIA)  • fatal pulmonary autoimmunity  • hepatosplenomegaly  **Validation:** lack of PD-1 on patient PBMCs  • extensive biochemical and genetic analysis of mutant PD1  • reduced production of IFNγ by PD1-def T cells in response to mycobacterial stimuli  • recapitulates previously-reported phenotype of *Pdcd1*-targetted mice | Table 4  Subtable 4 | [40] |
| ***ELF4***  (3 patients, 3 unrelated families) | XL | • Normal lymphocyte numbers | • Normal B cell numbers | • Normal levels of serum IgM, G, A  • reduced responses to live viral vaccines | • early onset mucosal autoinflammation/ IBD, fevers and ulcers  •  responded to IL-1, TNF or IL-12p40 blockade  • hyper inflammatory macrophages  **Validation:** mouse models, showed impaired expression of anti-inflammatory genes; developed IBD | Table 4  Subtable 5 | [41, 42] |
| ***TET2***  (3 patients, 2 unrelated families) | AR (LOF) | • 🡑 DN T cells  • 🡓Th1, Th17, Tfh cells | • 🡓Memory B cells; • impaired B-cell differentiation in vitro to plasma cells | • variable (hyper-/ hypogamma, 🡓 response to pneumococcal vaccine | • ALPS-like (🡑  sCD25, sFasL, IL10)  • recurrent viral infections, lymphadenopathy, hepatosplenomegaly, autoimmunity (cytopenias, B-cell lymphoma (EBV+ HL-like)  • failure to thrive, developmental delay  • EBV viremia  • DNA hypermethylation  • defective FAS-mediated apoptosis | Table 4  Subtable 7 | [43] |
| ***CEBPE***  (3 patients, 1 family) | AR (GOF) | • mild lymphopenia  • 🡓 naive, 🡑 T_EMRA_ cells | ND | ND | • Recurrent abdominal pain, aseptic fever, systemic inflammation; abscesses, ulceration, infections; mild bleeding diathesis.  • Autoinflammasome activation/ 🡑 IFN gene expression (auto-inflammation, immunodeficiency, neutrophil dysfunction)  **Validation**  • homozygous CEBPE variant affecting DNA binding domain, associated with autoinflammation/immunodeficiency  • showed altered chromatin occupancy of mutant CEBPE, and transcriptional changes, in patient cells targeting inflammasome and IFN-related genes | Table 4  Subtable 2 | [44] |
| ***IKZF1*** (IKAROS) GOF  (8 patients, 4 families) | AD GOF | • normal %’s total T cells  • 🡑 CD4/CD8 ratio, • 🡑 CD4+ T_EM_, CD8+ T_EMRA_ cells  DN T cells | • Normal/mild decrease in total B cells  • 🡑 trans B | • normal/ 🡑 IgG, IgA, 🡑 IgE; normal IgM  • autoAbs | • multiple autoimmune (diabetes, colitis, thyroiditis), allergic, lymphoproliferative features, plasma cell expansion (IgG4^+^), Evans Syndrome  • recurrent infections  **Validation**  • increased binding of mutant IKAROS to DNA/target genes | Table 4  Subtable 3 | [45] |
| ***CXCR2***  (6 patients, 5 families) | AR (LOF) | • normal numbers of CD4+ T cells,  • variable CD8+ T cells  • NK cells normal | • normal B cell numbers | • high serum IgG, IgA, IgM; | • profound neutropenia  • myelokathexis (3/6 pts)  • recurrent gingivitis, oral ulcers  • hypergammaglobulinemia  **Validation:** reduced/abolished expression of mutant CXCR2 on patient cells, impaired responses to CXCL8  • recapitulates phenotype of *Cxcr2*^-/-^ mice | Table 5  Subtable 1 | [46, 47] |
| ***TBX21*** (T-bet)  (1 patient) | AR (LOF) | • normal %’s total, CD4 and CD8 T cells, naïve and memory subsets,  • 🡓 NK cells  •  🡓 CXCR3^+^CCR6^-^ Th1 cells, CXCR3^+^ Tfh and CXCR3^+^ Treg CD4^+^T cells  •  🡑 immature NK •  🡓 iNKT, MAIT, Vδ2^+^ γδ T cells | • normal | • normal | • MSMD  • upper airway inflammation  • 🡓 IFN-γ and TNF-α production by T cell subsets (γδ T cells, MAIT cells, iNKT cells, NK cells, Vδ2^+^γδ, Vδ1^+^γδ, and CD4^+^ T cells  **Validation**  • biochemical and molecular analysis established the impact of this variant on Tbet function  • impaired production of IFNγ, TNFα by *TBX21*-mutant naïve CD4+ T cells under Th1 polarizing culture conditions in vitro  • Tbet-dependent functions restored in patient cells and cell lines by WT *TBX21* | Table 6  Subtable 1 | [48, 49] |
| ***IFNG***  (2 related patients [cousins]) | AR (LOF) | • Normal frequencies of T and NK cells;  • 🡑 proportions of naive CD4^+^ and CD8+ T cells; • 🡓 frequency of invariant iNKT | • Normal B cell frequencies  • 🡓 memory B cells (🡓IgA^+^/IgM^+^, 🡑 IgG^+^ memory  B cells) | • Normal | MSMD/BCG-osis  • no IFN-γ producing cells  **Validation**  • LOE and LOF allele  • biochemical and molecular analysis established impact of this variant on IFN-γ production from patient cells  •  no IFN-γ production by T and NK cells;  • impaired IFN-γ production by patient-derived *H saimiri-* immortalized T cells restored by introduction of WT *IFNG* | Table 6  Subtable 1 | [50] |
| ***NOS2***  (1 patient) | AR | • 🡓 CD4^+^ T cells;  • 🡓 NK cells (mostly all immature cells)  • normal CD8+ T | • 🡓 B cells | • specific Ab levels normal | • Severe susceptibility to CMV-induced disease; fatal  • pneumocystis pneumonia secondary to CMV  • apparent intact responses to infection with other herpes viruses (EBV, VZV, HSV)  **Validation:**  • confirmed functional defect in transfected cells; truncated NOS2 failed to induce nitrous oxide  **•** recapitulates susceptibility of *Nos2* deficient mice to murine CMV infection [51] (these mice are also susceptible to numerous other pathogens) | Table 6  Subtable 3 | [52] |
| ***ZNFX1***  (28 patients, 17 families) | AR | • Normal lymphocyte numbers  • intact T cell function |  |  | •  severe infections by RNA/DNA viruses  •  early-onset, systemic, severe, acute inflammation with major dysfunction of liver, brain, kidneys, lungs  • virally triggered inflammatory episodes (HLH)  • intermittent monocytosis, thrombocytopenia  •  hepatosplenomegaly, lymphadenopathy  • mycobacterial disease (BCGosis, disseminated TB)  • 11/15,1/4, 2/9 pts died in 3 different studies (14/28)  •  🡑 ISG in response to poly I/C | Table 6  Subtable 3 | [53-55] |
| ***SNORA31***  (5 patients, unrelated) | AD | • Normal | • Normal | • seropositive for IgG against many viruses | • Forebrain herpes simplex virus-1 (HSV1) encephalitis  **Validation:** susceptibility of human pluripotent stem cell  (hPSC)-derived cortical neurons from patients or hPSC-derived neurons from healthy donors but engineered to express variant *SNORA31* to HSV1 infection, corrected by exogenous IFN-β  • Incomplete penetrance | Table 6  Subtable 4 | [56] |
| ***ATG4A***  (1 patient) | AD | • Normal | • Normal | • Normal | • Mollaret’s meningitis (recurrent lymphocytic meningitis) due to HSV2  • History of multiple episodes of meningitis; HSV2^+^  **Validation**  • impaired HSV2-induced autophagy 🡒 increased viral replication and apoptosis of patient fibroblasts  • these defects were rescued by introduction of WT *ATG4* or *LC3B2* into patient fibroblasts | Table 6  Subtable 4 | [57] |
| ***MAP1LC3B2***  (1 patient) |  |  |  |  |  |  |  |
| ***MAPK8***  (3 patients, 1 family) | AD  (haplo-insufficiency) | • normal total T cells, CD4^+^ and CD8^+^ T subsets, NK cells  • 🡓 Th17 cells | • normal total B cells and subsets | • Normal | • chronic mucocutaneous candidiasis (CMC)  • connective tissue disorder (similar to Ehlers-Danlos syndrome)  • 🡓 Th17 cells ex vivo, in vitro  • 🡓 responses of fibroblasts to IL-17A, IL-17F  •  🡓 c-Jun/ATF-2-dependant TGF β signaling  **Validation**  • *MAKP8* variant LOE in HEK293 T, heterozygous patient cells  (🡓 Th17 cells ex vivo, in vitro; 🡓 fibroblast responses to IL-17A, IL-17F; 🡓 TGFβ signaling)  • defective responses of fibroblasts restored by WT MAPK8 | Table 6  Subtable 6 | [58] |
| ***TLR7***  (21 patients, many families) | XL |  | | | • Severe COVID19 infection  **Validation:**  • impaired responses to TLR7 ligands/SARS CoV2; reduced production of type 1 IFN | Table 6  Subtable 7 | [59-61] |
| ***TLR8***  (3 patients, 2 unrelated families) | XL GOF | • 🡑(mild) CD4+, CD8+ T cells, effector/memory subsets  • 🡓NK cells  • 🡓 RBCs, neutrophils, platelets,  • normal B cells/subsets, normal/low igG, 🡑IgM/IgA  🡓 pDCs | | | • early onset, severe chronic AIHA, neutropenia (post-infections),  • hepatosplenomegaly, lymphadenopathy  • progressive autoinflammatory disease (fevers, enteritis, arthritis, CNS vasculitis, IBD-like disease, ulcers)  • elevated proinﬂammatory serum cytokines  • fatal in 1 patient  **Validation:**  • increased pro-inflammatory responses (🡑 NFkB activation, 🡑 pro-inflammatory cytokines) of patient monocytes/iPSC-derived myeloid cells to TLR8 agonists  • reduced ability of mutant TLR8 to attenuate TLR7 signaling | Table 6  Subtable 7 | [62, 63] |
| ***TMEM173***  (6 patients, 4 unrelated families) | AR GOF | • 🡑 Ig levels | | | • failure to thrive  • early onset rash, fever, dyspnea, interstitial lung disease/pneumonitis, polyarthritis, autoAbs  • clubbing  • increased inflammatory markers, IFN gene signature  • phenocopy of SAVI due to AD GOF *TMEM173* variants  • 2 patients succumbed; 4 successfully treated with JAK inhibitors  **Validation:**  • increased activation of IFN-target genes in HEK293T cells by patient STING variant (ligand-independent) | Table 7  Subtable 1 | [64] |
| ***LSM11*** (2 siblings, 1 family) | AR (LOF) | Not reported | | | • Aicardi-Goutieres syndrome (AGS, type 1 IFN-opathy)  • increased inflammatory markers, IFN signaling in fibroblasts cells | Table 7  Subtable 1 | [65] |
| ***RNU7****-1* (16 patients, 11 unrelated families) |  |  |  |  |  |  |  |
| ***CDC42***  (15 patients; numerous kindreds) | AD | • normal/decreased T cell numbers,  • normal %CD4/CD8 but skewed differentiation | • Normal/B-lymphopenia | • variable (🡑🡓) IgM, G, A, E | • Neonatal onset: pancytopenia, fever, rash, hepatosplenomegaly, multisystemic inflammation, myelofibrosis/proliferation, HLH, enterocolitis  • recurrent gastrointestinal/respiratory tract infections;  • neurodevelopmental delay, FTT  • 🡑 serum levels of IL1, IL18, IFN-γ, ferritin, sCD25, CRP etc,  • Mutation affects actin function;  • treated with Anakinra/IFN-γ mAb  • 🡓 NK function (cytotoxicity), | Table 7  Subtable 1 | [66-72] |
| ***STAT2***  *(LOF*,* 3 patients; all deceased; 2 additional deceased sibs but not genotyped; 2 unrelated families) | AR (STAT2^R148^  LOF/regulation) | • low frequency of NK,  • 🡑 frequency of T cells (esp naïve), • normal NK degranulation | • Total B cell frequencies within range of age-matched controls  • slight 🡑 transitional and naïve B cells %’s | • low/normal | • Severe fatal early onset autoinflammation (skin ulceration, fever, seizures, intracranial calcification, multiorgan dysfunction, abnormal neurodevelopment; phenocopy of USP18 deficiency)  • 🡑 serum IFN-α, IL6, TNFα  • IFN-opathy gene signature (impaired regulation of late cellular responses to type 1 IFN),  **Validation**  **•** mutant *STAT2* alleles studied in *STAT2* deficient human cell line, and patient’s immortalized fibroblasts  • patient cells hyper-sensitive to IFN-α 🡒 prolonged JAK/STAT signaling/transcriptional activation  • mutant allele is homozygous LOF for binding to and trafficking of USP18, a negative regulator of type 1 IFN responses, to IFNαR2.  • 🡒🡑 late (not early) response to type 1 IFNs  • biochemical GOF of type 1 IFN signalling due to loss of regulatory activity of STAT2 | Table 7  Subtable 1 | [73, 74] |
| ***ATAD3A***  (8 patients, 7 unrelated families) | AD  (1 AR) |  |  |  | • predominantly neurological defects (development delay, spasticity)  • elevated ISG expression, increased serum type 1 IFNs  **Validation:** KD of *ATAD3A* in cell lines 🡒🡑 type IFN signaling, STING activation | Table 7  Subtable 1 | [75] |
| ***RIPK1***  (12 patients; 5 families) | AD | • Normal T and NK cell numbers  • low/normal CD4^+^ T cells  • normal/hi CD8^+^ T cells  • 🡑 DN T cells | • Normal B cells | ND | • Autoinflammatory disorder: regular/prolonged fevers, lymphadenopathy, spleno/hepatomegaly, ulcers, arthralgia, GI features,  • 🡑 inflamm markers, 🡑 pro-inflamm cytokines/gene signature; • responsive to Tocilizumab (not IL1/TNF blockade) | Table 7  Subtable 2 | [76, 77] |
| ***C2orf69***  (28 patients, 13 families) | AR |  |  |  | • Early onset of severe recurrent autoinflammation disorder,  • often fatal  •  Global developmental delay, (hypomyelinisation, leukoencephalopathy, microcephaly) with recurrent seizures, Muscle weakness  • liver dysfunction | Table 7  Subtable 3 | [78, 79] |
| ***NCKAP1L***  (9 patients; 7 families) | AR (LOF) | • normal T cell numbers  • 🡑 T_CM_, exhausted cells;  • possibly immature NK cells but intact function | • Normal B cells and naïve/memory subsets  • 🡑 CD21^lo^ cells | • Normal/🡑 Ig levels  • autoAbs | • Recurrent URTI, skin rashes/abscesses, ulcers,  • anti dsDNA Abs, SLE-like, lymphadenopathy, fever, HLH-like  • FTT  • immunodeficiency coupled with atopy, lymphoproliferation, hyperinflammation and cytokine overproduction (🡑 Th1)  • 🡓 T cell proliferation, cytoskeletal defects | Table 7  Subtable 3 | [80-82] |
| ***SYK***  (6 patients, 5 families) | AD GOF | • 🡓 CD4+ T cells  • ↑ CD8+ T cells  • ↑ CD4+ Th17, Th1 cells. | 🡓 memory B cells | • dysgamma   🡓IgM,  🡓IgG | • immune deficiency/recurrent infections  • multi-organ inflammatory disease (colitis, arthritis, dermatitis)  • inflammation in multiple tissues (gut, skin, CNS, lung, liver)  • diffuse large B cell lymphoma (2 pts)  • WBC counts, CRP  **Validation:** SYK variants extensively analyzed biochemically, found to increase phosphorylation, enhance downstream signaling  • KI (SYK-Ser544Tyr) mouse model recapitulated aspects of the human disease; partially treated with SYK inhibitor or WT HSCT | Table 7  Subtable 3 | [83] |
| ***HCK***  (1 patient) | AD GOF | • 🡑 production of inflammatory cytokines (IL-1β, IL-6, IL-8, TNF-α), reactive oxygen species. | | | • cutaneous vasculitis and chronic pulmonary inflammation/fibrosis  • inflammatory leukocyte infiltration of the lungs and skin  • anemia, hepatosplenomegaly, death due to respiratory failure  • clinical improvement with ruxolitinib  **Validation:**  • increased kinase activity of HCK mutant in vitro  • enhanced functions in transfected myeloid cell lines: 🡑 production of inflammatory cytokines (IL-1β, IL-6, IL-8, TNF-α), reactive oxygen species. | Table 7  Subtable 3 | [84] |
| ***PSMB9***  (3 patients, 3 families) | AD GOF | • Mild pancytopenia  • Intact T cell proliferation in vitro  • ↓ IgG and B cell numbers  ↓TREC and KREC, indicating T & B cell defects.  ↓ monocytes, CD8 T, and γδ T cells  ↓ activity of NK cells | | | •  severe autoinflammatory phenotype (neonatal-onset fever, a chilblain-like skin rash, myositis, severe pulmonary hypertension, basal ganglia calcification), periodic inflammatory exacerbation.  • immunodeficiency  • Clinical features partially overlapped with PRAAS  • elevated levels of inflammatory cytokines (IL-6, IL-18, IP-10, IFNα) and liver enzymes in blood and CSF (IFNα)  •  hyperactivation of IFN-α, pSTAT1.  **Validation:**  • reduced proteasome activities  •  mouse model expressing one of the heterozygous variants recapitulated proteasome defects and immunodeficiency | Table 7  Subtable 3 | [85, 86] |
| ***IKBKG* (NEMO exon 5 deletion** (NEMO-Δex5, 5 patients) | XL | • progressive B-cell lymphopenia  • hypogammaglobulinemia | | | • fever, skin rash, systemic autoinflammation, infections, CNS involvement, panniculitis, uveitis, hepatosplenomegaly, ectodermal dysplasia in some patients  **Validation:**  • mutant NEMO lacked exon 5 (NEMO-Δex5), failed to bind TBK1  • NEMO-Δex5 protein stabilized IKKi, increasing type 1 IFN production | Table 7  Subtable 3 | [87-89] |
| ***TBK1*** (4 patients, 3 families) | AR | Not reported | | | • chronic systemic autoinflammation (polyarthritis, vasculitis,  • delayed neurocognitive development  • treated with TNF inhibitors  **Validation:**  • autoinflammation driven by TNF-induced RIPK1-dependent cell death | Table 7  Subtable 3 | [90] |
| ***MECOM*** (many) | AD LOF | • Variable degrees of congenital thrombocytopenia/pancytopenia;  • B-cell deficiency | | | • bone marrow failure, radioulnar synostosis, clinodactyly, cardiac and renal malformations,  **Validation:**  Defective binding of MECOM/EVI1 to cognate binding site | Table 9 | [91, 92] |
| ***TLR8***  (5 patients, 5 unrelated families) | XL  (somatic GOF mutations) | • 🡑(mild) CD4+, CD8+ T cells, effector/memory subsets  • 🡓NK cells  • 🡓 RBCs, neutrophils, platelets,  • normal B cells/subsets, normal/low igG, 🡑IgM/IgA  🡓 pDCs | | | • severe cytopenias (AIHA, neutropenia, thrombocytopenia),  • hepatosplenomegaly, lymphadenopathy  • recurrent infections (pneumonia, otitis, fungi)  • hypocellular bone marrow  • elevated proinﬂammatory serum cytokines  • fatal in 1 patient  **Validation:**  • increased pro-inflammatory responses (🡑 NFkB activation, 🡑 pro-inflammatory cytokines) of patient monocytes/iPSC-derived myeloid cells to TLR8 agonists | Table 10 | [62] |
| ***UBA1*** (>25 patients) | XL  (somatic LOF mutations) | • 🡓 peripheral lymphocyte counts  • loss of immature B cells, non-classical and intermediate monocyte populations | | | • late adulthood onset treatment-refractory inflammatory syndrome (VEXAS)  • fevers, cytopenias, dysplastic bone marrow,  • neutrophilic cutaneous and pulmonary inflammation, interstitial nephritis, chondritis, cardiac involvement, stroke, and vasculitis  • most patients have an inflammatory syndrome (relapsing polychondritis, Sweet syndrome, polyarteritis nodosa, giant-cell arteritis) or a hematologic condition (MDS, multiple myeloma)  • often fatal  • Dysregulated proinflammatory neutrophil activation, high inflammatory markers in patient serum  **Validation:**  **•**overexpression of mutant allele favored production of a catalytically deficient UBA1  • defective ubiquitylation in patient mutant monocytes  • CRISPR-Cas9 *Uba1*–deficient zebrafish model recapitulates the phenotype of systemic inflammation | Table 10 | [93] |

| **Novel phenocopies of Inborn Errors of Immunity** | | | | |
| --- | --- | --- | --- | --- |
| **Disease** | **Mechanisms of disease pathogenesis** | **Associated/clinical features** | **Table** | **Refs** |
| Severe COVID-19 | • high levels of neutralizing anti-type 1 IFNs (IFNα, IFNω) autoAbs | • severe, life-threatening infection with SARS-CoV-2 | Table 10 | [94-98] |

***Abbreviations:*** AR: autosomal recessive; AD: autosomal dominant; AID: activation-induced cytidine deaminase; CSR: class switch recombination; SHM: somatic hypermutation; MDS: myelodysplastic syndrome; LOE: loss of expression; LOF: loss of function; GOF: gain of function; DN: dominant negative; MSMD: Mendelian susceptibility to mycobacterial disease; HLH: hemophagocytic lymphohistiocytosis; FTT: failure to thrive; hPSC: human pluripotent stem cells; iPSC: induced pluripotent stem cells; CMV: cytomegalovirus

**References**

1. Bousfiha A, Jeddane L, Picard C, Al-Herz W, Ailal F, Chatila T et al. Human Inborn Errors of Immunity: 2019 Update of the IUIS Phenotypical Classification. J Clin Immunol. 2020;40(1):66-81. doi:10.1007/s10875-020-00758-x.

2. Tangye SG, Al-Herz W, Bousfiha A, Chatila T, Cunningham-Rundles C, Etzioni A et al. Human Inborn Errors of Immunity: 2019 Update on the Classification from the International Union of Immunological Societies Expert Committee. J Clin Immunol. 2020;40(1):24-64. doi:10.1007/s10875-019-00737-x.

3. Lev A, Lee YN, Sun G, Hallumi E, Simon AJ, Zrihen KS et al. Inherited SLP76 deficiency in humans causes severe combined immunodeficiency, neutrophil and platelet defects. J Exp Med. 2021;218(3). doi:10.1084/jem.20201062.

4. Yamazaki Y, Urrutia R, Franco LM, Giliani S, Zhang K, Alazami AM et al. PAX1 is essential for development and function of the human thymus. Sci Immunol. 2020;5(44). doi:10.1126/sciimmunol.aax1036.

5. Paganini I, Sestini R, Capone GL, Putignano AL, Contini E, Giotti I et al. A novel PAX1 null homozygous mutation in autosomal recessive otofaciocervical syndrome associated with severe combined immunodeficiency. Clin Genet. 2017;92(6):664-8. doi:10.1111/cge.13085.

6. Almutairi A, Wallace JG, Jaber F, Alosaimi MF, Jones J, Sallam MTH et al. Severe combined immunodeficiency caused by inositol-trisphosphate 3-kinase B (ITPKB) deficiency. J Allergy Clin Immunol. 2020. doi:10.1016/j.jaci.2020.01.014.

7. Delmonte OM, Bergerson JRE, Kawai T, Kuehn HS, McDermott DH, Cortese I et al. SASH3 variants cause a novel form of X-linked combined immunodeficiency with immune dysregulation. Blood. 2021;138(12):1019-33. doi:10.1182/blood.2020008629.

8. Labrador-Horrillo M, Franco-Jarava C, Garcia-Prat M, Parra-Martinez A, Antolin M, Salgado-Perandres S et al. Case Report: X-Linked SASH3 Deficiency Presenting as a Common Variable Immunodeficiency. Front Immunol. 2022;13:881206. doi:10.3389/fimmu.2022.881206.

9. Verheijen J, Wong SY, Rowe JH, Raymond K, Stoddard J, Delmonte OM et al. Defining a new immune deficiency syndrome: MAN2B2-CDG. J Allergy Clin Immunol. 2020;145(3):1008-11. doi:10.1016/j.jaci.2019.11.016.

10. Bainter W, Platt CD, Park SY, Stafstrom K, Wallace JG, Peters ZT et al. Combined immunodeficiency due to a mutation in the gamma1 subunit of the coat protein I complex. J Clin Invest. 2021;131(3). doi:10.1172/JCI140494.

11. Hetemaki I, Kaustio M, Kinnunen M, Heikkila N, Keskitalo S, Nowlan K et al. Loss-of-function mutation in IKZF2 leads to immunodeficiency with dysregulated germinal center reactions and reduction of MAIT cells. Sci Immunol. 2021;6(65):eabe3454. doi:10.1126/sciimmunol.abe3454.

12. Shahin T, Kuehn HS, Shoeb MR, Gawriyski L, Giuliani S, Repiscak P et al. Germline biallelic mutation affecting the transcription factor Helios causes pleiotropic defects of immunity. Sci Immunol. 2021;6(65):eabe3981. doi:10.1126/sciimmunol.abe3981.

13. Hadjadj J, Aladjidi N, Fernandes H, Leverger G, Magerus-Chatinet A, Mazerolles F et al. Pediatric Evans syndrome is associated with a high frequency of potentially damaging variants in immune genes. Blood. 2019;134(1):9-21. doi:10.1182/blood-2018-11-887141.

14. Shahin T, Mayr D, Shoeb MR, Kuehn HS, Hoeger B, Giuliani S et al. Identification of Germline Monoallelic Mutations in IKZF2 in Patients with Immune Dysregulation. Blood Adv. 2021. doi:10.1182/bloodadvances.2021006367.

15. Bainter W, Lougaris V, Wallace JG, Badran Y, Hoyos-Bachiloglu R, Peters Z et al. Combined immunodeficiency with autoimmunity caused by a homozygous missense mutation in inhibitor of nuclear factor B kinase alpha (IKKalpha). Sci Immunol. 2021;6(63):eabf6723. doi:10.1126/sciimmunol.abf6723.

16. Mace EM, Paust S, Conte MI, Baxley RM, Schmit MM, Patil SL et al. Human NK cell deficiency as a result of biallelic mutations in MCM10. J Clin Invest. 2020. doi:10.1172/JCI134966.

17. Baxley RM, Leung W, Schmit MM, Matson JP, Yin L, Oram MK et al. Bi-allelic MCM10 variants associated with immune dysfunction and cardiomyopathy cause telomere shortening. Nat Commun. 2021;12(1):1626. doi:10.1038/s41467-021-21878-x.

18. Beziat V, Tavernier SJ, Chen YH, Ma CS, Materna M, Laurence A et al. Dominant-negative mutations in human IL6ST underlie hyper-IgE syndrome. J Exp Med. 2020;217(6). doi:10.1084/jem.20191804.

19. Monies D, Abouelhoda M, Assoum M, Moghrabi N, Rafiullah R, Almontashiri N et al. Lessons Learned from Large-Scale, First-Tier Clinical Exome Sequencing in a Highly Consanguineous Population. Am J Hum Genet. 2019;104(6):1182-201. doi:10.1016/j.ajhg.2019.04.011.

20. Chen YH, Grigelioniene G, Newton PT, Gullander J, Elfving M, Hammarsjo A et al. Absence of GP130 cytokine receptor signaling causes extended Stuve-Wiedemann syndrome. J Exp Med. 2020;217(3). doi:10.1084/jem.20191306.

21. Wu B, Rice L, Shrimpton J, Lawless D, Walker K, Carter C et al. Biallelic mutations in calcium release activated channel regulator 2A (CRACR2A) cause a primary immunodeficiency disorder. Elife. 2021;10. doi:10.7554/eLife.72559.

22. Kaustio M, Nayebzadeh N, Hinttala R, Tapiainen T, Astrom P, Mamia K et al. Loss of DIAPH1 causes SCBMS, combined immunodeficiency, and mitochondrial dysfunction. J Allergy Clin Immunol. 2021;148(2):599-611. doi:10.1016/j.jaci.2020.12.656.

23. Yamashita M, Kuehn HS, Okuyama K, Okada S, Inoue Y, Mitsuiki N et al. A variant in human AIOLOS impairs adaptive immunity by interfering with IKAROS. Nat Immunol. 2021;22(7):893-903. doi:10.1038/s41590-021-00951-z.

24. Kuehn HS, Chang J, Yamashita M, Niemela JE, Zou C, Okuyama K et al. T and B cell abnormalities, pneumocystis pneumonia, and chronic lymphocytic leukemia associated with an AIOLOS defect in patients. J Exp Med. 2021;218(12). doi:10.1084/jem.20211118.

25. Beziat V, Rapaport F, Hu J, Titeux M, Bonnet des Claustres M, Bourgey M et al. Humans with inherited T cell CD28 deficiency are susceptible to skin papillomaviruses but are otherwise healthy. Cell. 2021;184(14):3812-28 e30. doi:10.1016/j.cell.2021.06.004.

26. Park H, Staehling K, Tsang M, Appleby MW, Brunkow ME, Margineantu D et al. Disruption of Fnip1 reveals a metabolic checkpoint controlling B lymphocyte development. Immunity. 2012;36(5):769-81. doi:10.1016/j.immuni.2012.02.019.

27. Siggs OM, Stockenhuber A, Deobagkar-Lele M, Bull KR, Crockford TL, Kingston BL et al. Mutation of Fnip1 is associated with B-cell deficiency, cardiomyopathy, and elevated AMPK activity. Proc Natl Acad Sci U S A. 2016;113(26):E3706-15. doi:10.1073/pnas.1607592113.

28. Niehues T, Ozgur TT, Bickes M, Waldmann R, Schoning J, Brasen J et al. Mutations of the gene FNIP1 associated with a syndromic autosomal recessive immunodeficiency with cardiomyopathy and pre-excitation syndrome. Eur J Immunol. 2020;50(7):1078-80. doi:10.1002/eji.201948504.

29. Saettini F, Poli C, Vengoechea J, Bonanomi S, Orellana JC, Fazio G et al. Absent B cells, agammaglobulinemia, and hypertrophic cardiomyopathy in Folliculin Interacting Protein 1 deficiency. Blood. 2020. doi:10.1182/blood.2020006441.

30. Le Coz C, Nguyen DN, Su C, Nolan BE, Albrecht AV, Xhani S et al. Constrained chromatin accessibility in PU.1-mutated agammaglobulinemia patients. J Exp Med. 2021;218(7). doi:10.1084/jem.20201750.

31. Thian M, Hoeger B, Kamnev A, Poyer F, Kostel Bal S, Caldera M et al. Germline biallelic PIK3CG mutations in a multifaceted immunodeficiency with immune dysregulation. Haematologica. 2020. doi:10.3324/haematol.2019.231399.

32. Takeda AJ, Maher TJ, Zhang Y, Lanahan SM, Bucklin ML, Compton SR et al. Human PI3Kgamma deficiency and its microbiota-dependent mouse model reveal immunodeficiency and tissue immunopathology. Nat Commun. 2019;10(1):4364. doi:10.1038/s41467-019-12311-5.

33. Kury P, Staniek J, Wegehaupt O, Janowska I, Eckenweiler M, Korinthenberg R et al. Agammaglobulinemia with normal B-cell numbers in a patient lacking Bob1. J Allergy Clin Immunol. 2021;147(5):1977-80. doi:10.1016/j.jaci.2021.01.027.

34. Kuhny M, Forbes LR, Cakan E, Vega-Loza A, Kostiuk V, Dinesh RK et al. Disease-associated CTNNBL1 mutation impairs somatic hypermutation by decreasing nuclear AID. J Clin Invest. 2020. doi:10.1172/JCI131297.

35. Yeh TW, Okano T, Naruto T, Yamashita M, Okamura M, Tanita K et al. APRIL-dependent life-long plasmacyte maintenance and immunoglobulin production in humans. J Allergy Clin Immunol. 2020. doi:10.1016/j.jaci.2020.03.025.

36. Kalinichenko A, Perinetti Casoni G, Dupre L, Trotta L, Huemer J, Galgano D et al. RhoG deficiency abrogates cytotoxicity of human lymphocytes and causes hemophagocytic lymphohistiocytosis. Blood. 2021;137(15):2033-45. doi:10.1182/blood.2020008738.

37. Lee PY, Platt CD, Weeks S, Grace RF, Maher G, Gauthier K et al. Immune dysregulation and Multisystem Inflammatory Syndrome in Children (MIS-C) in individuals with haploinsufficiency of SOCS1. J Allergy Clin Immunol. 2020. doi:10.1016/j.jaci.2020.07.033.

38. Thaventhiran JED, Lango Allen H, Burren OS, Rae W, Greene D, Staples E et al. Whole-genome sequencing of a sporadic primary immunodeficiency cohort. Nature. 2020;583(7814):90-5. doi:10.1038/s41586-020-2265-1.

39. Hadjadj J, Castro CN, Tusseau M, Stolzenberg MC, Mazerolles F, Aladjidi N et al. Early-onset autoimmunity associated with SOCS1 haploinsufficiency. Nat Commun. 2020;11(1):5341. doi:10.1038/s41467-020-18925-4.

40. Ogishi M, Yang R, Aytekin C, Langlais D, Bourgey M, Khan T et al. Inherited PD-1 deficiency underlies tuberculosis and autoimmunity in a child. Nat Med. 2021;27(9):1646-54. doi:10.1038/s41591-021-01388-5.

41. Tyler PM, Bucklin ML, Zhao M, Maher TJ, Rice AJ, Ji W et al. Human autoinflammatory disease reveals ELF4 as a transcriptional regulator of inflammation. Nat Immunol. 2021;22(9):1118-26. doi:10.1038/s41590-021-00984-4.

42. Sun G, Qiu L, Yu L, An Y, Ding Y, Zhou L et al. Loss of Function Mutation in ELF4 Causes Autoinflammatory and Immunodeficiency Disease in Human. J Clin Immunol. 2022. doi:10.1007/s10875-022-01243-3.

43. Stremenova Spegarova J, Lawless D, Mohamad SMB, Engelhardt KR, Doody G, Shrimpton J et al. Germline TET2 loss of function causes childhood immunodeficiency and lymphoma. Blood. 2020;136(9):1055-66. doi:10.1182/blood.2020005844.

44. Goos H, Fogarty CL, Sahu B, Plagnol V, Rajamaki K, Nurmi K et al. Gain-of-function CEBPE mutation causes noncanonical autoinflammatory inflammasomopathy. J Allergy Clin Immunol. 2019;144(5):1364-76. doi:10.1016/j.jaci.2019.06.003.

45. Hoshino A, Boutboul D, Zhang Y, Kuehn HS, Hadjadj J, Ozdemir N et al. Gain-of-function IKZF1 variants in humans cause immune dysregulation associated with abnormal T/B cell late differentiation. Sci Immunol. 2022;7(69):eabi7160. doi:10.1126/sciimmunol.abi7160.

46. Marin-Esteban V, Youn J, Beaupain B, Jaracz-Ros A, Barlogis V, Fenneteau O et al. Biallelic CXCR2 loss-of-function mutations define a distinct congenital neutropenia entity. Haematologica. 2021. doi:10.3324/haematol.2021.279254.

47. Auer PL, Teumer A, Schick U, O'Shaughnessy A, Lo KS, Chami N et al. Rare and low-frequency coding variants in CXCR2 and other genes are associated with hematological traits. Nat Genet. 2014;46(6):629-34. doi:10.1038/ng.2962.

48. Yang R, Mele F, Worley L, Langlais D, Rosain J, Benhsaien I et al. Human T-bet Governs Innate and Innate-like Adaptive IFN-gamma Immunity against Mycobacteria. Cell. 2020;183(7):1826-47 e31. doi:10.1016/j.cell.2020.10.046.

49. Yang R, Weisshaar M, Mele F, Benhsaien I, Dorgham K, Han J et al. High Th2 cytokine levels and upper airway inflammation in human inherited T-bet deficiency. J Exp Med. 2021;218(8). doi:10.1084/jem.20202726.

50. Kerner G, Rosain J, Guerin A, AlKhabaz A, Oleaga-Quintas C, Rapaport F et al. Inherited human IFNgamma deficiency underlies mycobacterial disease. J Clin Invest. 2020. doi:10.1172/JCI135460.

51. Noda S, Tanaka K, Sawamura S, Sasaki M, Matsumoto T, Mikami K et al. Role of nitric oxide synthase type 2 in acute infection with murine cytomegalovirus. J Immunol. 2001;166(5):3533-41. doi:10.4049/jimmunol.166.5.3533.

52. Drutman SB, Mansouri D, Mahdaviani SA, Neehus AL, Hum D, Bryk R et al. Fatal Cytomegalovirus Infection in an Adult with Inherited NOS2 Deficiency. N Engl J Med. 2020;382(5):437-45. doi:10.1056/NEJMoa1910640.

53. Vavassori S, Chou J, Faletti LE, Haunerdinger V, Opitz L, Joset P et al. Multisystem inflammation and susceptibility to viral infections in human ZNFX1 deficiency. J Allergy Clin Immunol. 2021;148(2):381-93. doi:10.1016/j.jaci.2021.03.045.

54. Le Voyer T, Neehus AL, Yang R, Ogishi M, Rosain J, Alroqi F et al. Inherited deficiency of stress granule ZNFX1 in patients with monocytosis and mycobacterial disease. Proc Natl Acad Sci U S A. 2021;118(15). doi:10.1073/pnas.2102804118.

55. Alawbathani S, Westenberger A, Ordonez-Herrera N, Al-Hilali M, Al Hebby H, Alabbas F et al. Biallelic ZNFX1 variants are associated with a spectrum of immuno-hematological abnormalities. Clin Genet. 2022;101(2):247-54. doi:10.1111/cge.14081.

56. Lafaille FG, Harschnitz O, Lee YS, Zhang P, Hasek ML, Kerner G et al. Human SNORA31 variations impair cortical neuron-intrinsic immunity to HSV-1 and underlie herpes simplex encephalitis. Nat Med. 2019;25(12):1873-84. doi:10.1038/s41591-019-0672-3.

57. Hait AS, Olagnier D, Sancho-Shimizu V, Skipper KA, Helleberg M, Larsen SM et al. Defects in LC3B2 and ATG4A underlie HSV2 meningitis and reveal a critical role for autophagy in antiviral defense in humans. Sci Immunol. 2020;5(54). doi:10.1126/sciimmunol.abc2691.

58. Li J, Ritelli M, Ma CS, Rao G, Habib T, Corvilain E et al. Chronic mucocutaneous candidiasis and connective tissue disorder in humans with impaired JNK1-dependent responses to IL-17A/F and TGF-beta. Sci Immunol. 2019;4(41). doi:10.1126/sciimmunol.aax7965.

59. Asano T, Boisson B, Onodi F, Matuozzo D, Moncada-Velez M, Maglorius Renkilaraj MRL et al. X-linked recessive TLR7 deficiency in ~1% of men under 60 years old with life-threatening COVID-19. Sci Immunol. 2021;6(62). doi:10.1126/sciimmunol.abl4348.

60. van der Made CI, Simons A, Schuurs-Hoeijmakers J, van den Heuvel G, Mantere T, Kersten S et al. Presence of Genetic Variants Among Young Men With Severe COVID-19. JAMA. 2020;324(7):663-73. doi:10.1001/jama.2020.13719.

61. Abolhassani H, Vosughimotlagh A, Asano T, Landegren N, Boisson B, Delavari S et al. X-Linked TLR7 Deficiency Underlies Critical COVID-19 Pneumonia in a Male Patient with Ataxia-Telangiectasia. J Clin Immunol. 2021. doi:10.1007/s10875-021-01151-y.

62. Aluri J, Bach A, Kaviany S, Chiquetto Paracatu L, Kitcharoensakkul M, Walkiewicz MA et al. Immunodeficiency and bone marrow failure with mosaic and germline TLR8 gain of function. Blood. 2021;137(18):2450-62. doi:10.1182/blood.2020009620.

63. Fejtkova M, Sukova M, Hlozkova K, Skvarova Kramarzova K, Rackova M, Jakubec D et al. TLR8/TLR7 dysregulation due to a novel TLR8 mutation causes severe autoimmune hemolytic anemia and autoinflammation in identical twins. Am J Hematol. 2022;97(3):338-51. doi:10.1002/ajh.26452.

64. Lin B, Berard R, Al Rasheed A, Aladba B, Kranzusch PJ, Henderlight M et al. A novel STING1 variant causes a recessive form of STING-associated vasculopathy with onset in infancy (SAVI). J Allergy Clin Immunol. 2020;146(5):1204-8 e6. doi:10.1016/j.jaci.2020.06.032.

65. Uggenti C, Lepelley A, Depp M, Badrock AP, Rodero MP, El-Daher MT et al. cGAS-mediated induction of type I interferon due to inborn errors of histone pre-mRNA processing. Nat Genet. 2020;52(12):1364-72. doi:10.1038/s41588-020-00737-3.

66. Verboon JM, Mahmut D, Kim AR, Nakamura M, Abdulhay NJ, Nandakumar SK et al. Infantile Myelofibrosis and Myeloproliferation with CDC42 Dysfunction. J Clin Immunol. 2020. doi:10.1007/s10875-020-00778-7.

67. Lam MT, Coppola S, Krumbach OHF, Prencipe G, Insalaco A, Cifaldi C et al. A novel disorder involving dyshematopoiesis, inflammation, and HLH due to aberrant CDC42 function. J Exp Med. 2019;216(12):2778-99. doi:10.1084/jem.20190147.

68. Gernez Y, de Jesus AA, Alsaleem H, Macaubas C, Roy A, Lovell D et al. Severe autoinflammation in 4 patients with C-terminal variants in cell division control protein 42 homolog (CDC42) successfully treated with IL-1beta inhibition. J Allergy Clin Immunol. 2019;144(4):1122-5 e6. doi:10.1016/j.jaci.2019.06.017.

69. Bucciol G, Pillay B, Casas-Martin J, Delafontaine S, Proesmans M, Lorent N et al. Systemic Inflammation and Myelofibrosis in a Patient with Takenouchi-Kosaki Syndrome due to CDC42 Tyr64Cys Mutation. J Clin Immunol. 2020. doi:10.1007/s10875-020-00742-5.

70. Bekhouche B, Tourville A, Ravichandran Y, Tacine R, Abrami L, Dussiot M et al. A toxic palmitoylation of Cdc42 enhances NF-kappaB signaling and drives a severe autoinflammatory syndrome. J Allergy Clin Immunol. 2020. doi:10.1016/j.jaci.2020.03.020.

71. He T, Huang Y, Ling J, Yang J. A New Patient with NOCARH Syndrome Due to CDC42 Defect. J Clin Immunol. 2020;40(4):571-5. doi:10.1007/s10875-020-00786-7.

72. Szczawinska-Poplonyk A, Ploski R, Bernatowska E, Pac M. A Novel CDC42 Mutation in an 11-Year Old Child Manifesting as Syndromic Immunodeficiency, Autoinflammation, Hemophagocytic Lymphohistiocytosis, and Malignancy: A Case Report. Front Immunol. 2020;11:318. doi:10.3389/fimmu.2020.00318.

73. Gruber C, Martin-Fernandez M, Ailal F, Qiu X, Taft J, Altman J et al. Homozygous STAT2 gain-of-function mutation by loss of USP18 activity in a patient with type I interferonopathy. J Exp Med. 2020;217(5). doi:10.1084/jem.20192319.

74. Duncan CJA, Thompson BJ, Chen R, Rice GI, Gothe F, Young DF et al. Severe type I interferonopathy and unrestrained interferon signaling due to a homozygous germline mutation in STAT2. Sci Immunol. 2019;4(42). doi:10.1126/sciimmunol.aav7501.

75. Lepelley A, Della Mina E, Van Nieuwenhove E, Waumans L, Fraitag S, Rice GI et al. Enhanced cGAS-STING-dependent interferon signaling associated with mutations in ATAD3A. J Exp Med. 2021;218(10). doi:10.1084/jem.20201560.

76. Tao P, Sun J, Wu Z, Wang S, Wang J, Li W et al. A dominant autoinflammatory disease caused by non-cleavable variants of RIPK1. Nature. 2020;577(7788):109-14. doi:10.1038/s41586-019-1830-y.

77. Lalaoui N, Boyden SE, Oda H, Wood GM, Stone DL, Chau D et al. Mutations that prevent caspase cleavage of RIPK1 cause autoinflammatory disease. Nature. 2020;577(7788):103-8. doi:10.1038/s41586-019-1828-5.

78. Wong HH, Seet SH, Maier M, Gurel A, Traspas RM, Lee C et al. Loss of C2orf69 defines a fatal autoinflammatory syndrome in humans and zebrafish that evokes a glycogen-storage-associated mitochondriopathy. Am J Hum Genet. 2021;108(7):1301-17. doi:10.1016/j.ajhg.2021.05.003.

79. Lausberg E, Giesselmann S, Dewulf JP, Wiame E, Holz A, Salvarinova R et al. C2orf69 mutations disrupt mitochondrial function and cause a multisystem human disorder with recurring autoinflammation. J Clin Invest. 2021;131(12). doi:10.1172/JCI143078.

80. Cook SA, Comrie WA, Poli MC, Similuk M, Oler AJ, Faruqi AJ et al. HEM1 deficiency disrupts mTORC2 and F-actin control in inherited immunodysregulatory disease. Science. 2020;369(6500):202-7. doi:10.1126/science.aay5663.

81. Salzer E, Zoghi S, Kiss MG, Kage F, Rashkova C, Stahnke S et al. The cytoskeletal regulator HEM1 governs B cell development and prevents autoimmunity. Sci Immunol. 2020;5(49). doi:10.1126/sciimmunol.abc3979.

82. Castro CN, Rosenzwajg M, Carapito R, Shahrooei M, Konantz M, Khan A et al. NCKAP1L defects lead to a novel syndrome combining immunodeficiency, lymphoproliferation, and hyperinflammation. J Exp Med. 2020;217(12). doi:10.1084/jem.20192275.

83. Wang L, Aschenbrenner D, Zeng Z, Cao X, Mayr D, Mehta M et al. Gain-of-function variants in SYK cause immune dysregulation and systemic inflammation in humans and mice. Nat Genet. 2021;53(4):500-10. doi:10.1038/s41588-021-00803-4.

84. Kanderova V, Svobodova T, Borna S, Fejtkova M, Martinu V, Paderova J et al. Early-onset pulmonary and cutaneous vasculitis driven by constitutively active SRC-family kinase HCK. J Allergy Clin Immunol. 2021. doi:10.1016/j.jaci.2021.07.046.

85. Kataoka S, Kawashima N, Okuno Y, Muramatsu H, Miwata S, Narita K et al. Successful treatment of a novel type I interferonopathy due to a de novo PSMB9 gene mutation with a Janus kinase inhibitor. J Allergy Clin Immunol. 2021;148(2):639-44. doi:10.1016/j.jaci.2021.03.010.

86. Kanazawa N, Hemmi H, Kinjo N, Ohnishi H, Hamazaki J, Mishima H et al. Heterozygous missense variant of the proteasome subunit beta-type 9 causes neonatal-onset autoinflammation and immunodeficiency. Nat Commun. 2021;12(1):6819. doi:10.1038/s41467-021-27085-y.

87. de Jesus AA, Hou Y, Brooks S, Malle L, Biancotto A, Huang Y et al. Distinct interferon signatures and cytokine patterns define additional systemic autoinflammatory diseases. J Clin Invest. 2020;130(4):1669-82. doi:10.1172/JCI129301.

88. Hegazy S, Marques MC, Canna SW, Goldbach-Mansky R, de Jesus AA, Reyes-Mugica M et al. NEMO-NDAS: A Panniculitis in the Young Representing an Autoinflammatory Disorder in Disguise. Am J Dermatopathol. 2022. doi:10.1097/DAD.0000000000002144.

89. Lee Y, Wessel AW, Xu J, Reinke JG, Lee E, Kim SM et al. Genetically programmed alternative splicing of NEMO mediates an autoinflammatory disease phenotype. J Clin Invest. 2022;132(6). doi:10.1172/JCI128808.

90. Taft J, Markson M, Legarda D, Patel R, Chan M, Malle L et al. Human TBK1 deficiency leads to autoinflammation driven by TNF-induced cell death. Cell. 2021;184(17):4447-63 e20. doi:10.1016/j.cell.2021.07.026.

91. Niihori T, Ouchi-Uchiyama M, Sasahara Y, Kaneko T, Hashii Y, Irie M et al. Mutations in MECOM, Encoding Oncoprotein EVI1, Cause Radioulnar Synostosis with Amegakaryocytic Thrombocytopenia. Am J Hum Genet. 2015;97(6):848-54. doi:10.1016/j.ajhg.2015.10.010.

92. Germeshausen M, Ancliff P, Estrada J, Metzler M, Ponstingl E, Rutschle H et al. MECOM-associated syndrome: a heterogeneous inherited bone marrow failure syndrome with amegakaryocytic thrombocytopenia. Blood Adv. 2018;2(6):586-96. doi:10.1182/bloodadvances.2018016501.

93. Beck DB, Ferrada MA, Sikora KA, Ombrello AK, Collins JC, Pei W et al. Somatic Mutations in UBA1 and Severe Adult-Onset Autoinflammatory Disease. N Engl J Med. 2020. doi:10.1056/NEJMoa2026834.

94. Bastard P, Rosen LB, Zhang Q, Michailidis E, Hoffmann HH, Zhang Y et al. Autoantibodies against type I IFNs in patients with life-threatening COVID-19. Science. 2020;370(6515). doi:10.1126/science.abd4585.

95. Bastard P, Gervais A, Le Voyer T, Rosain J, Philippot Q, Manry J et al. Autoantibodies neutralizing type I IFNs are present in ~4% of uninfected individuals over 70 years old and account for ~20% of COVID-19 deaths. Sci Immunol. 2021;6(62). doi:10.1126/sciimmunol.abl4340.

96. Abers MS, Rosen LB, Delmonte OM, Shaw E, Bastard P, Imberti L et al. Neutralizing type-I interferon autoantibodies are associated with delayed viral clearance and intensive care unit admission in patients with COVID-19. Immunol Cell Biol. 2021;99(9):917-21. doi:10.1111/imcb.12495.

97. Troya J, Bastard P, Planas-Serra L, Ryan P, Ruiz M, de Carranza M et al. Neutralizing Autoantibodies to Type I IFNs in >10% of Patients with Severe COVID-19 Pneumonia Hospitalized in Madrid, Spain. J Clin Immunol. 2021;41(5):914-22. doi:10.1007/s10875-021-01036-0.

98. Solanich X, Rigo-Bonnin R, Gumucio VD, Bastard P, Rosain J, Philippot Q et al. Pre-existing Autoantibodies Neutralizing High Concentrations of Type I Interferons in Almost 10% of COVID-19 Patients Admitted to Intensive Care in Barcelona. J Clin Immunol. 2021;41(8):1733-44. doi:10.1007/s10875-021-01136-x.
